# The molecular basis of the genesis of basal tone in internal anal sphincter

**DOI:** 10.1038/ncomms11358

**Published:** 2016-04-22

**Authors:** Cheng-Hai Zhang, Pei Wang, Dong-Hai Liu, Cai-Ping Chen, Wei Zhao, Xin Chen, Chen Chen, Wei-Qi He, Yan-Ning Qiao, Tao Tao, Jie Sun, Ya-Jing Peng, Ping Lu, Kaizhi Zheng, Siobhan M. Craige, Lawrence M. Lifshitz, John F. Keaney Jr, Kevin E. Fogarty, Ronghua ZhuGe, Min-Sheng Zhu

**Affiliations:** 1 State Key Laboratory of Pharmaceutical Biotechnology and Model Animal Research Center and MOE Key Laboratory of Model Animal for Disease Study, Nanjing University, Nanjing 210061, China; 2 Department of Microbiology and Physiological Systems, University of Massachusetts Medical School, Worcester, Massachusetts 01605, USA; 3 CAM-SU Genomic Resource Center, Soochow University, Suzhou 215123, China; 4 Department of Medicine, University of Massachusetts Medical School, Worcester, Massachusetts 01655, USA; 5 Program in Molecular Medicine, University of Massachusetts Medical School, Worcester, Massachusetts 01605, USA; 6 Innovation Center for Cardiovascular Disorders, Beijing 100029, China

## Abstract

Smooth muscle sphincters exhibit basal tone and control passage of contents through organs such as the gastrointestinal tract; loss of this tone leads to disorders such as faecal incontinence. However, the molecular mechanisms underlying this tone remain unknown. Here, we show that deletion of myosin light-chain kinases (MLCK) in the smooth muscle cells from internal anal sphincter (IAS-SMCs) abolishes basal tone, impairing defecation. Pharmacological regulation of ryanodine receptors (RyRs), L-type voltage-dependent Ca^2+^ channels (VDCCs) or TMEM16A Ca^2+^-activated Cl^−^ channels significantly changes global cytosolic Ca^2+^ concentration ([Ca^2+^]_i_) and the tone. TMEM16A deletion in IAS-SMCs abolishes the effects of modulators for TMEM16A or VDCCs on a RyR-mediated rise in global [Ca^2+^]_i_ and impairs the tone and defecation. Hence, MLCK activation in IAS-SMCs caused by a global rise in [Ca^2+^]_i_ via a RyR-TMEM16A-VDCC signalling module sets the basal tone. Targeting this module may lead to new treatments for diseases like faecal incontinence.

## Abstract (web summary)

The molecular basis of the basal tone generated by internal anal sphincters (IAS) is largely unknown. Here, the authors show that the tone arises from a global rise in intracellular Ca^2+^ in smooth muscle cells via a Ryanodine receptor-TMEM16A-L-type Ca^2+^ channel-MLC kinase pathway, suggesting a potential therapy for IAS motility disorders.

## Graphical abstract

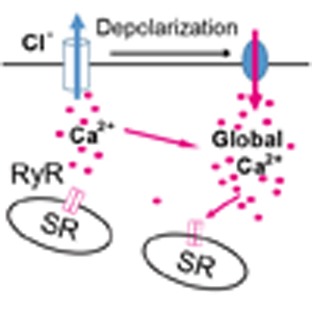

The human body, and those of other mammals, contains up to 50 sphincters, ring-shaped structures encircling an opening or passage in hollow organs such as the intestine and the bladder. These sphincters control the entrance of material into, or the release of contents from, these organs, and participate in a variety of biological functions essential for homeostasis^1^. Dysfunction in the sphincters, either structurally or functionally, can have severe consequences leading to diseases/disorders including gastroesophageal reflux disease, achalasia, gastroparesis, dysphagia, recurrent episodes of pancreatitis or biliary pain, faecal incontinence and urinary incontinence^1^. Healthy sphincters open transiently but, in the basal state, remain closed and therefore require constant force generation from the smooth muscle cells that make up sphincters. It is thus of fundamental importance to determine the molecular and cellular mechanisms that dictate sphincter smooth muscle contraction at rest (basal tone formation).

The internal anal sphincter (IAS) located at the end of the gastrointestinal tract, has served as a prototypical model to understand basal tone genesis in sphincters. A significant number of *in vitro* and *in vivo* experiments have indicated that the basal tone of IAS is independent of extrinsic nerve and hormone stimulation^2^, but instead is an intrinsic property of the sphincter smooth muscle itself. Smooth muscle force generation results from the cross-bridge movement of myosin and actin filaments on 20-kDa myosin regulatory light-chain phosphorylation (p-RLC)^3^. The amount of p-RLC is controlled by the balanced activation of Ca^2+^/calmodulin-dependent MLC kinase (MLCK) and Ca^2+^-independent MLC phosphatase (MLCP). MLCP consists of three subunits including a regulatory 110–130 kDa subunit, called the myosin-targeting subunit of MLCP (MYPT1), which anchors MLCP to p-RLC. MLCP can be phosphorylated by activation of small GTPase RhoA and Rho-associated, coiled-coil containing serine/threonine kinase (ROCK)^4^. Based on pharmacological and biochemical evidence, it has been suggested that a lower activity of MLCP as a result of ROCK-mediated phosphorylation of p^Thr696^-MYPT1 may be responsible for the basal tone in IAS (refs 5, 6, 7).

In this study, we use smooth-muscle-specific MYPT1 knockout mice to directly test this hypothesis. We find that the basal tone of IAS from the knockout mice is the same as that from wild-type mice. We, therefore, also test a new hypothesis that Ca^2+^-mediated MLCK activation is required for the IAS basal tone. We find that the basal tone in IAS from MLCK knockout mice is essentially abolished and these mice give rise to larger faeces, a sign of impaired faecal continence. By directly examining Ca^2+^ signals and ion channel activity, we further find that Ca^2+^-releasing ryanodine receptors/channels (RyRs), TMEM16A Ca^2+^-activated Cl^−^ (Cl_Ca_) channels and L-type voltage-dependent Ca^2+^ channels (VDCCs) form a module which generates a global rise in Ca^2+^, and that pharmacologically altering any one of the three channels can severely impair IAS basal tone (to the same degree as MLCK deletion). Moreover, genetic deletion of TMEM16A in IAS smooth muscle cells (IAS-SMCs) severely impairs both the RyR-mediated Ca^2+^ rise and the basal tone, and results in wider and longer faeces. Our results hence demonstrate that MLCK activation by a RyR-TMEM16A Cl_Ca_ channel-L-type VDCC signalling cascade in the IAS-SMCs is required for basal tone formation and maintenance, and is essential for faecal continence.

## Results

### MLCK is required for basal tone and evoked contraction in IAS

**Figure 1:**
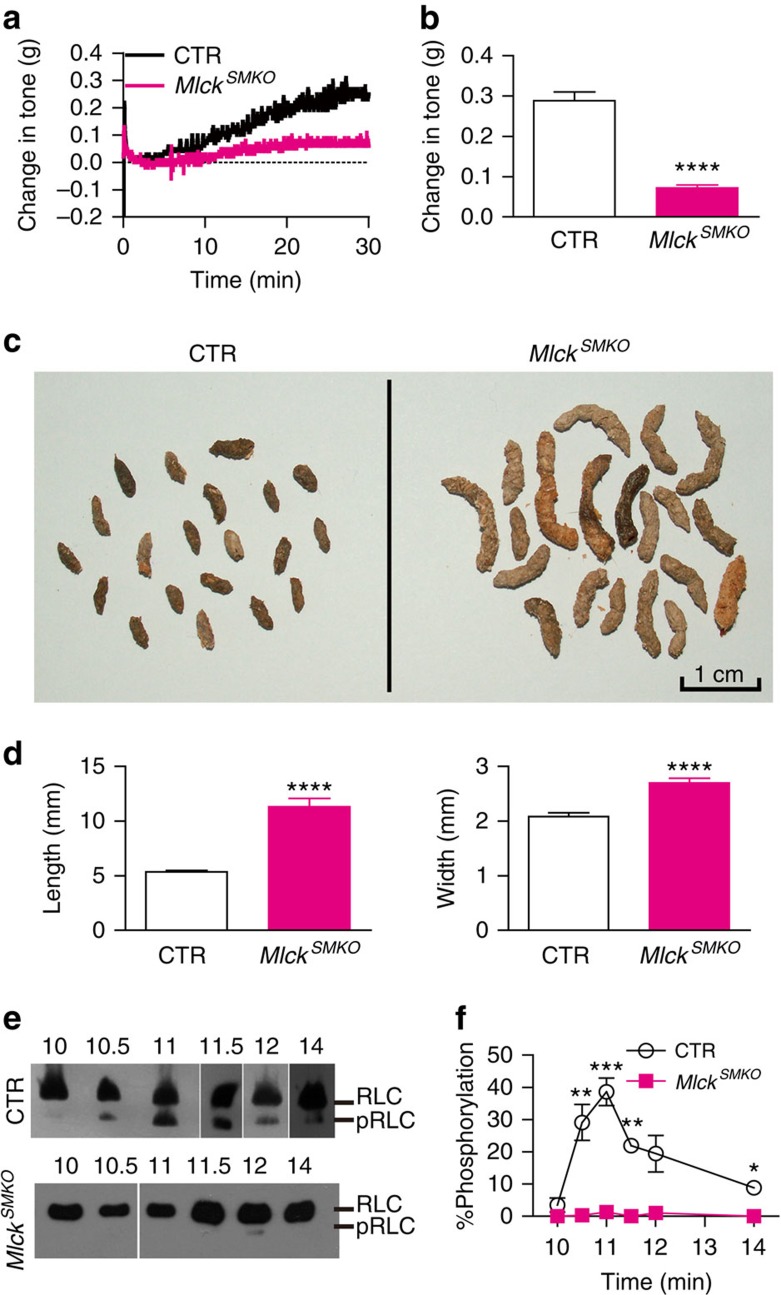
MLCK and RLC phosphorylation are required for the basal tone in IAS. (**a**) Time courses of changes in force after application of 0.5 g tension in CTR and *Mlck*^*SMKO*^ mice. (**b**) Summarized data showing much smaller IAS tone in *Mlck*^*SMKO*^ mice than in control (mean±s.e.m., CTR *n*=7, *Mlck*^*SMKO*^ *n*=5, ****P<0.0001 by two-tailed Student's *t*-test). (**c**) Faeces from CTR mice and *Mlck*^*SMKO*^ mice after 20 days of tamoxifen treatment. (**d**) The length (left) and width (right) of faeces were increased in *Mlck*^*SMKO*^ mice compared with the controls. Bars represent mean±s.e.m., *n*=20, ****P<0.0001 by two-tailed Student's *t*-test. (**e**) Examples of RLC phosphorylation during the process of spontaneous tone generation in IAS from CTR and *Mlck*^*SMKO*^ mice. (**f**) Quantification of p-RLC during basal tone generation in CTR and *Mlck*^*SMKO*^ IAS. Bars represent mean±s.e.m., *n*=3, *P<0.05, **P<0.01, ***P<0.001 by two-tailed Student's *t*-test.

IAS is a phenotypical sphincter consisting mainly of circular smooth muscle. To assay the basal tone, we employed a standard protocol^8^,^9^ in which excised mouse IAS strips had a 0.5-g ‘load' applied to them. In response to this load, the IAS gradually generated force (Fig. 1a, black trace; Supplementary Fig. 1 for the tone measurement). The slow development of the tone may reflect the unique smooth muscle arrangement in IAS, that is, it is divided into ‘minibundles' separated by connective tissue septa without electrical couplings^10^,^11^, so that the IAS is not readily synchronized as a whole. To directly examine the role of MYPT1 in the IAS basal tone^5^,^6^,^7^, we assessed the effect of MYPT1 deletion on this tone using MYPT1 knockout mice^12^. The development (time to 50% of the plateau) of the IAS basal tone was no different between MYPT1 knockout mice (8.4±0.62 min, *n*=12) and their littermate controls (10±0.97 min, *n*=10; *P*>0.05, by two-tailed Student's *t*-test), neither was the amplitude of the tone (MYPT1 knockout: 0.24±0.03 g, *n*=15; control: 0.22±0.02 g, *n*=13, *P*>0.05 by two-tailed Student's *t*-test) (Supplementary Fig. 2). Our genetic evidence thus indicates that p-RLC regulated by MYPT1-mediated MLCP activity may be not necessary for basal tone generation. This implies that p-RLC, regulated by MLCK, may be a determinant of IAS basal tone.

To directly test whether MLCK is required for IAS basal tone formation, we analysed the tone generation and contractile properties of IAS from tamoxifen inducible MLCK-deficient mice (*Mlck*^*SMKO*^)^13^. We found that 20 days after tamoxifen injection^14^, MLCK in IAS from *Mlck*^*SMKO*^ mice was reduced by 70–98%. For the phenotypic analysis, other than where noted, only the IAS tissue in which MLCK was decreased by >95% was used (Supplementary Fig. 3a). But, as shown in Fig. 1a,b, MLCK deletion completely inhibited the basal tone (*Mlck*^*SMKO*^: 0.07±0.01 g; control (CTR): 0.29 ±0.02 g, *P*<0.0001 by two-tailed Student's *t*-test; Fig. 1b). Moreover, after the 18th day post tamoxifen induction, mouse faeces became softer and larger. At the 20th day, the length of the faeces from *Mlck*^*SMKO*^ mice increased from 5.4±0.1 to 11.3±0.8 mm (*P*<0.0001 by two-tailed Student's *t*-test, *n*=20), and the diameter increased from 2.1±0.1 to 2.7±0.1 mm (*P*<0.0001 by two-tailed Student's *t*-test, *n*=20) (Fig. 1c,d). These results suggest that MLCK deletion abolishes the basal tone, likely resulting in weaker compacting capability and slight rectoanal incontinence^15^,^16^.

To determine whether the suppression of the IAS basal tone in *Mlck*^*SMKO*^ mice was due to a decrease in regulatory light-chain (RLC) phosphorylation, we measured p-RLC in *Mlck*^*SMKO*^ mice and their controls. As shown in Fig. 1e,f, IAS muscle strips from the controls generated phosphorylated myosin light chain during tone development. Although the time course of p-RLC level was not strictly correlated with the time course of basal tone, p-RLC level was always above its basal level during tone development and maintenance. Significantly p-RLC in IAS from *Mlck*^*SMKO*^ was not detectable during the same time frame.

It is known that MLCK is required for phasic smooth muscle contraction induced by contractile agonists^13^. To determine whether this is also the case in sphincter smooth muscle, we examined the dependency of agonist-induced contraction on MLCK and RLC phosphorylation. On stimulation with KCl and bethanechol , IAS smooth muscle from the control mice displayed a robust contraction with prolonged tension (Supplementary Fig. 3b), whereas IAS from *Mlck*^*SMKO*^ mice developed much weaker contraction (26 and 28% of the controls with KCl and bethanechol, respectively) (Supplementary Fig. 3b,c). Moreover, the IAS from the *Mlck*^*SMKO*^ mice showed a significantly lower level of RLC phosphorylation at different time points on stimulation with KCl or bethanechol (Supplementary Fig. 3d,e).

The decreases in both the basal tone and the evoked contraction in the MLCK-deficient IAS cells were not due to a structural change in IAS because with standard H&E staining, no apparent changes in structure or cell morphology in IAS from these *Mlck*^*SMKO*^ mice were detected (Supplementary Fig. 4). Neither could they be explained by compensatory changes in the cyclic guanosine monophosphate (cGMP)/cGMP-dependent kinase (PKG), protein kinase C (PKC) and RhoA/ROCK signalling pathways because, as shown in Supplementary Fig. 5, the expressions of soluble guanylate cyclase (sGC), PKG, integrin-linked kinase (ILK), PKC and CPI-17 were not changed significantly in IAS between *Mlck*^*SMKO*^ mice and control mice. Together, the above results demonstrate that both the generation of basal tone and evoked contraction in IAS requires MLCK activation and RLC phosphorylation.

### Ion channel activation is required for basal tone in IAS

**Figure 2:**
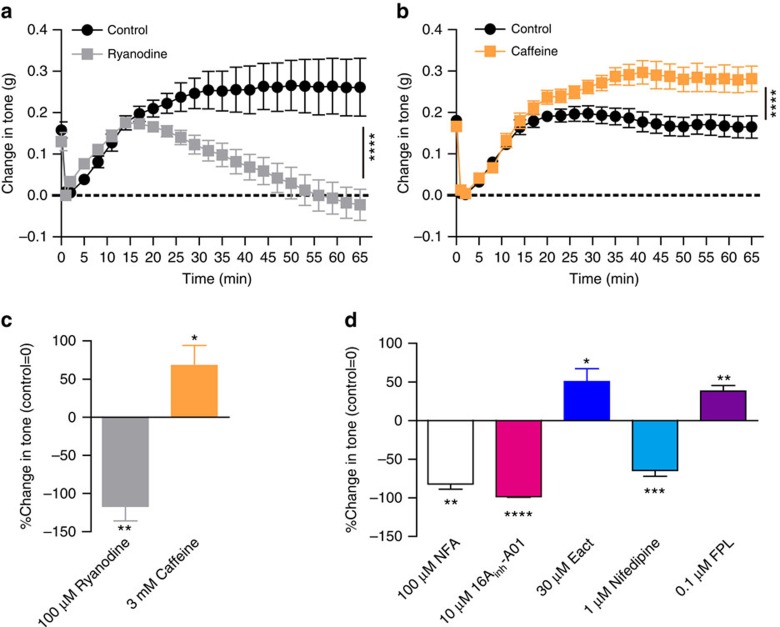
RyRs, Cl_Ca_channels and VDCCs are essential for the basal tone in IAS. (**a**) Ryanodine (100 μM) significantly decreased the spontaneous tone in IAS. Bars represent mean±s.e.m., control *n*=5, ryanodine *n*=6, ****P<0.0001 by analysis of variance (ANOVA) comparing the sustained phases. (**b**) Caffeine (3 mM) increased the tone in IAS. Bars represent mean±s.e.m., *n*=7, ****P<0.0001 by ANOVA comparing the sustained phases. (**c**) Summarized results on the IAS tone affected by 100μM ryanodine (*n*=6), 3 mM caffeine (*n*=8). Bars depict mean±s.e.m., *P<0.05, **P<0.01 by paired two-tailed Student's *t*-test. (**d**) Summarized results on the IAS tone affected by 100 μM niflumic acid (NFA; *n*=8), 10 μM 16A_inh-_A01 (*n*=4), 30 μM E_act_ (*n*=5), 1 μM nifedipine (*n*=8) and 0.1 μM FPL64176 (*n*=5). Bars depict mean±s.e.m., *P<0.05, **P<0.01, ***P<0.001, ****P<0.0001 by paired two-tailed Student's *t*-test.

The requirement of MLCK activation in the basal tone formation in IAS prompted us to search for the Ca^2+^ signalling mechanism essential for this tone. Given the myogenic nature of the basal tone and the autonomic activation of ryanodine receptors (RyRs) at resting Ca^2+^ concentration ([Ca^2+^]_i_), we hypothesized that RyRs could be a critical component of this essential Ca^2+^ signal. To examine this possibility, we investigated the effects of (RyR inhibitor) ryanodine and (RyR agonist) caffeine on IAS basal tone. As shown in Fig. 2, ryanodine (100 μM) fully blocked the tone (Fig. 2a,c). On the other hand, caffeine (3 mM) increased the maximal level of the basal tone ∼68% over the control (*P*<0.05 by paired two-tailed Student's *t*-test; Fig. 2b,c). These results indicate that RyRs alone, or in conjunction with other molecules, can generate the calcium signal essential for basal tone formation in IAS. L-type VDCCs blockers reverse the IAS basal tone of various animals^9^,^17^. We confirmed this in mice since (1) mouse IAS-SMCs exhibit typical L-type Ca^2+^ currents, which can be blocked by nifedipine, a specific L-type VDCC blocker (Supplementary Fig. 6); (2) nifedipine fully inhibited the basal tone, and FPL64176, a specific L-type VDCC agonist,^18^,^19^ enhanced the tone (Fig. 2d; Supplementary Fig. 7e,g). The above results concerning RyRs and VDCCs raise a possibility that RyR-mediated Ca^2+^ events functionally couple with L-type VDCCs to control the basal tone. In several smooth muscles, Cl_Ca_ channels act as the mediator coupling RyR and L-type VDCC activity, that is, a local or global rise in Ca^2+^ due to RyR opening activates Cl_Ca_ channels, which depolarizes the membrane and turns on L-type VDCCs (refs 20, 21, 22). To investigate whether this mechanism underlies the IAS tone formation, we examined changes in the tone by 16A_inh-_A01 (a specific blocker of TMEM16A Cl_Ca_ channels^23^) and niflumic acid (a non-specific blocker of Cl_Ca_ channels^24^). We found that niflumic acid and 16A_inh_-A01 dose-dependently reversed the basal tone (Supplementary Fig. 7a–d). Niflumic acid at 100 μM and 16A_inh_-A01 at 10 μM fully reversed the basal tone (Fig. 2d; Supplementary Fig. 7a–d), similar to the effect of MLCK deletion. Moreover, E_act_, a newly developed activator of the TMEM16A Cl_Ca_ channel^25^, potentiated the basal tone (Fig. 2d; Supplementary Fig. 7f). Since the inhibitors for RyRs, the Cl_Ca_ channels and L-type VDCCs can all fully reverse the basal tone while their agonists can potentiate the tone, we propose that these three channels form a signalling module which sets this tone in IAS.

### A RyR-mediated rise in global [Ca^2+^]_i_ contracts IAS-SMCs

**Figure 3:**
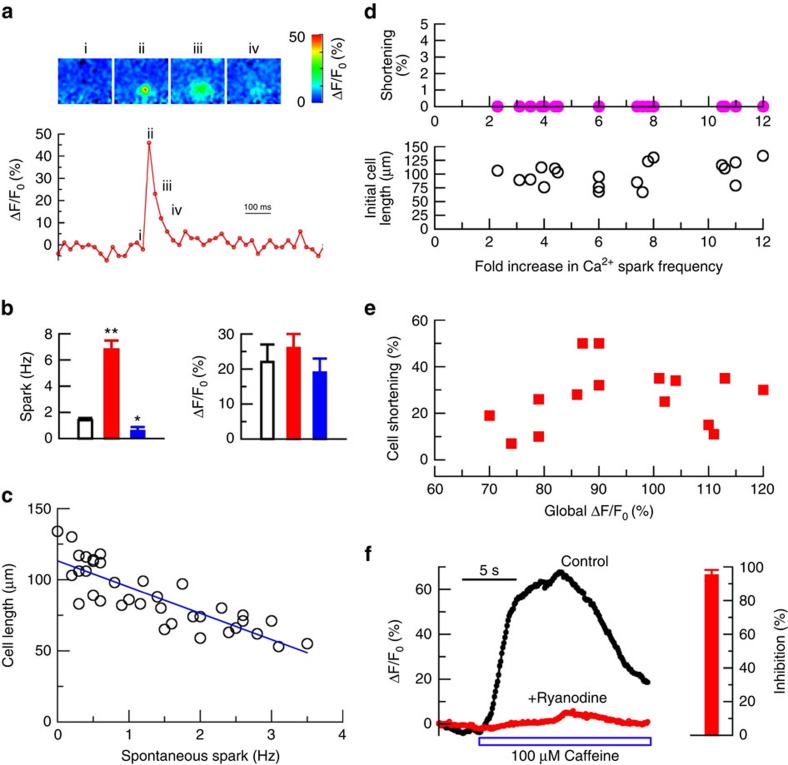
RyR-mediated global [Ca^2+^]_i_, but not Ca^2+^ sparks, contracts IAS-SMCs. (**a**) Top panels show the spatiotemporal evolution of a single Ca^2+^ spark in an IAS-SMC, and the Roman numerals correspond to the time marked on the trace of time course of ΔF/F0 (%) (a proxy for the change in [Ca^2+^]_i_ at the epicentre pixel (333 × 333 nm) of this spark. (**b**) Effects of caffeine (180 μM, red bars) and ryanodine (100 μM, blue bars) on Ca^2+^ sparks. Bars represent mean±s.e.m., *n*=20 cells for caffeine, and *n*=4 cells for ryanodine. *P<0.05; **P<0.01 by two-tailed Student's *t*-test. (**c**) Calcium spark frequency was inversely correlated with cell length (Pearson correlation coefficient: −0.8398, *P*<0.0001 by two-tailed Student's *t*-test; *n*=36). (**d**) Ca^2+^ spark activity was increased by caffeine (30, 200 or 500 μM) but did not associate with any detectable shortening (upper panel) of cells with different initial lengths (lower panel; Pearson correlation coefficient: 0.3409, *P*>0.05 by two-tailed Student's *t*-test; *n*=19). (**e**) An increase in global [Ca^2+^]_i_ by 1 or 10 mM caffeine did cause cell shortening by 27.0±3.4% on average (*P*<0.01 by the Student's *t*-test; *n*=15) although no correlation exists between the magnitude of the caffeine-induced global [Ca^2+^]i and amount of cell shortening (Pearson correlation coefficient: 0.1596, *P*>0.05 by the Student's *t*-test; *n*=15). (**f**) Ryanodine (100 μM) blocked the caffeine-induced calcium increase. The bar chart on the right is the mean±s.e.m. Per cent inhibition by ryanodine for six cells.

Having established that the RyR-Cl_Ca_ channel-VDCC module controls the basal tone of IAS, we next studied the nature of the Ca^2+^ signalling generated by activation of these channels in IAS-SMCs. Since RyRs generate spontaneous Ca^2+^ sparks in a variety of smooth muscle cells^26^,^27^,^28^, these Ca^2+^ events may directly regulate IAS smooth muscle contraction. To test this possibility, we examined the relationship between Ca^2+^ sparks and cell length using isolated single IAS-SMCs. We found that freshly isolated IAS-SMCs generated spontaneous localized Ca^2+^ events (Fig. 3a) at a frequency of 1.4±0.2 Hz (*n*=36; Fig. 3b). These events can be classified as Ca^2+^ sparks because caffeine (180 μM) increased their activity by almost eightfold (*n*=10) while ryanodine at 100 μM suppressed them by 62%±22 (*n*=4) (Fig. 3b). Interestingly, as shown in Fig. 3c, there was an inverse relationship between the spark frequency and cell length, that is, the higher the frequency, the shorter the cell length. This inverse relationship raises a possibility that Ca^2+^ sparks may be a causal signal for cell shortening. If this is the case, we should observe that an increase in Ca^2+^ spark activity by other means shortens IAS-SMCs. To assess this possibility, we used caffeine to evoke Ca^2+^ sparks. In IAS-SMCs, caffeine elicited Ca^2+^ sparks at a concentration as low as 10 μM, which is about one-tenth the concentration needed in other types of smooth muscle cells^27^ (Supplementary Fig. 8). Interestingly, caffeine at concentrations that only increase Ca^2+^ sparks did not induce the shortening of cells with different original lengths (Fig. 3d). Only at the level which caused both a burst of Ca^2+^ sparks and a rise in global [Ca^2+^] did caffeine cause cell shortening (Fig. 3e). This caffeine-induced rise in global [Ca^2+^]i was mediated by RyR since ryanodine could essentially block the Ca^2+^ response evoked by caffeine (Fig. 3f). These results indicate that a RyR-mediated rise in global [Ca^2+^]_i_, and not the RyR-mediated Ca^2+^ sparks, regulate the shortening of IAS-SMCs.

### Cl_Ca_ channels and VDCCs contribute to the RyR-mediated global [Ca^2+^]_i_

**Figure 4:**
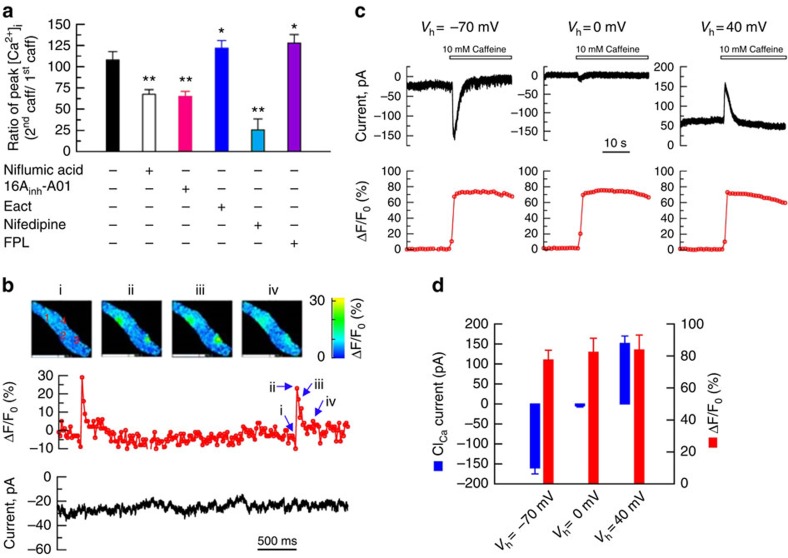
RyRs, Cl_Ca_ channels and VDCCs form a signaling module in IAS-SMCs. (**a**) Niflumic acid (100 μM), 16A_inh-_A01 (10 μM) and nifedipine (1 μM) partially inhibited, while E_act_ (30 μM) and FPL64176 (0.1 μM) potentiated, a caffeine (1 mM)-induced calcium increase. Data were calculated as the ratio of increase in Ca^2+^ by a second caffeine pulse over the first caffeine pulse. For each group, cells were pretreated with the modulator for ∼3 min before the application of the second caffeine pulse. Bars represent mean±s.e.m., *n*=3–8, *P<0.05; **P<0.01 versus caffeine alone group by two-tailed Student's *t*-test. (**b**) Individual sparks do not activate any Cl^−^ currents. Top panel shows the spatiotemporal evolution of two Ca^2+^ sparks (sites marked 1 and 3) in an IAS-SMC, below it is the associated time course for site 3 calculated as ΔF/F0 (%), that is, the change in [Ca^2+^]i from the spark's epicentre pixel, 330 × 330 nm, divided by its value from time 0. Images are displaying ΔF/F0 (%). Roman numerals on the time course are the time points of the four images on the top panel from site 3. Sites 2 and 4 displayed sparks at times not shown in this analysis. The lowest panel is the membrane current associated with these Ca^2+^ sparks. Note that none of the events is associated with membrane current changes, that is, no STICs (that is, spontaneous transient inward currents) were evoked by Ca^2+^ sparks. (**c**) Caffeine (10 mM) increased global Ca^2+^ and Cl_Ca_ currents. The currents reversed at E_Cl_ (∼0 mV), which indicates that the currents detected at −70 and +40 mV are due to the opening of Cl_Ca_ channels. (**d**) Summarized results for the 10 mM caffeine-induced global Ca^2+^ increases and their corresponding Cl_Ca_ currents at −70, 0 and +40 mV. Bars represent mean±s.e.m., *n*=4 cells, one from each of four mice.

Since RyRs, Cl_Ca_ channels and L-type VDCCs form a signalling module to control the basal tone (Fig. 2), and a RyR-mediated rise in global [Ca^2+^]_i_ is required to induce shortening of IAS-SMCs (Fig. 3), we asked whether Cl_Ca_ channels and L-type VDCCs contribute to the RyR-mediated rise in global [Ca^2+^]i. In the absence of extracellular Ca^2+^, caffeine increased [Ca^2+^]_i_ to a smaller extent (ΔF/F_0_: 108±23%, *n*=10) than in the presence of extracellular Ca^2+^ ((ΔF/F_0_: 159±28%, *n*=12)), indicating that in addition to Ca^2+^ release from ryanodine-sensitive Ca^2+^ stores, caffeine also induced Ca^2+^ influx. To determine the potential roles of the Cl_Ca_ channel and L-type VDCCs in this Ca^2+^ influx we compared the caffeine-induced Ca^2+^ rise in control cells with the rise produced when modulators of Cl_Ca_ channels or L-type VDCCs were present. Figure 4a shows that niflumic acid (100 μM), 16A_inh-_A01 (10 μM) and nifedipine (1 μM) inhibited the caffeine-induced increase in global [Ca^2+^]_i_ by 33, 35 and 74.0%, respectively. E_act_ (1μM) and FPL64176 (1 μM) enhanced the caffeine-induced rise in [Ca^2+^]_i_ by 28 and 20%, respectively. These results demonstrated that activation of RyRs can turn on Cl_Ca_ channels and L-type VDCCs, resulting in a global rise in [Ca^2+^]_i_.

### A global rise in [Ca^2+^]_i_ activates Cl_Ca_ currents in IAS-SMCs

To directly examine whether an increase in [Ca^2+^]_i_ can activate Cl_Ca_ channels, we examined the relationship between Ca^2+^ signals and Cl_Ca_ currents by simultaneously measuring [Ca^2+^]_i_ with high- speed imaging and membrane currents with the patch-clamp technique^28^. In several types of smooth muscle cells, Ca^2+^ sparks activate Cl_Ca_ channels to generate spontaneous transient inward currents (STICs)^29^. To our surprise, in mouse IAS-SMCs, Ca^2+^ sparks did not associate with any detectable currents when the membrane was clamped at voltages more negative than the reversal potential for Cl^−^ (Fig. 4b, *n*=5), indicating they do not activate STICs. We then assessed whether an increase in global [Ca^2+^]_i_ could elicit Cl_Ca_ currents. To raise global [Ca^2+^]_i_, we stimulated the cells with caffeine (10 mM) and recorded the membrane current at different voltages. As shown in Fig. 4c, on stimulation with caffeine, the IAS-SMC generated an inward current when held at −70 mV. This inward current was determined to result from the opening of Cl_Ca_ channels, because at the reversal potential for Cl^−^ (that is, 0 mV), the same cell failed to generate current in response to the same caffeine stimulation. At +40 mV, the cell produced a markedly outward current, as would be predicted if this current was a Cl^−^ current. Finally, caffeine raised the global [Ca^2+^]_i_ to the same level at three different holding potentials (Fig. 4c,d), indicating the difference in Cl^−^ currents at different holding potentials is not due to the variation in [Ca^2+^]_i_. The cells that generated these Cl^−^ currents are authentic smooth muscle cells as the IAS cells with positive green fluorescent protein (GFP) from α-smooth muscle actin-GFP mice produced similar currents in response to caffeine (Supplementary Fig. 9). In conclusion, these results argue that a rise in global [Ca^2+^]_i_ via RyR activation can activate Cl_Ca_ channels in IAS-SMCs.

### Ion channel expression in IAS-SMCs

It is well established that RyRs underlie Ca^2+^ sparks, and *Cav1* encodes L-type VDCCs in smooth muscle. PCR with reverse transcription (RT–PCR) detected three types of *Ryrs* with the dominant expression of *Ryr1* (Supplementary Fig. 10a), and of *Cav1* (that is, *Cav1.1*, *Cav1.2* and *Cav1.3*; Supplementary Fig. 10b) in IAS-SMCs. The TMEM16 family (that is, TMEM16A and TMEM16B) was recently found to function as Cl_Ca_ channels in several cell types^30^,^31^,^32^,^33^,^34^,^35^. We therefore decided to determine whether TMEM16A and/or TMEM16B are also expressed in IAS cells. With RT–PCR, we detected the transcripts of *Tmem16a* (Supplementary Fig. 10c). Interestingly, we failed to detect *Tmem16b* in IAS tissue (Supplementary Fig. 10d). An immunohistochemistry assay showed the colocalization between TMEM16A and Myh11, a specific smooth muscle marker, in IAS-SMCs, particularly in its inner layer (Supplementary Fig. 10e).

### Knockout of TMEM16A impairs the basal tone in IAS

**Figure 5:**
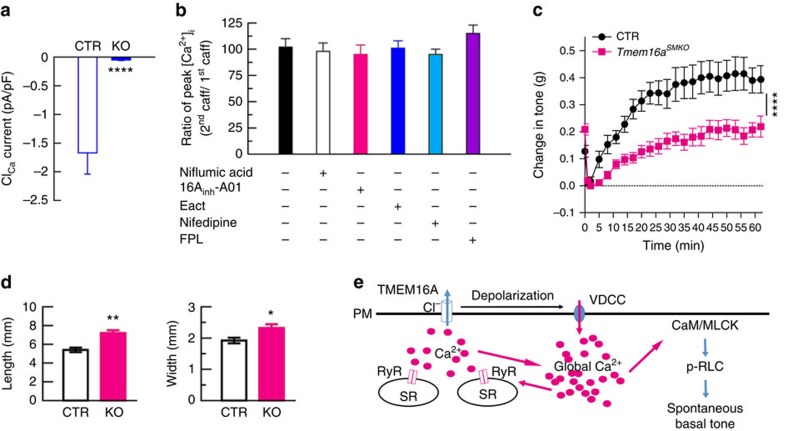
TMEM16A deletion impairs IAS smooth muscle function and faecal defecation. (**a**) Caffeine (10 mM)-induced Cl_Ca_ currents at the holding potential of −70 mV in IAS-SMCs from control and *Tmem16a*^*SMKO*^ mice. ****P<0.0001 KO versus CTR by two-tailed Student's *t*-test, *n*=14 for CTR and *n*=12 for KO (An additional five cells did not generated detectable Cl_Ca_ currents and were excluded in this calculation). (**b**) Effects of Cl_Ca_ channel and L-type Ca^2+^ channel modulators on caffeine-induced Ca^2+^ rise in IAS-SMCs from *Tmem16a*^*SMKO*^ mice. Experiments were carried out with a protocol as described in Fig. 4a. *n*=7–10 cells. (**c**) Basal tone in *Tmem16a*^*SMKO*^ mice is significantly reduced compared with CTR mice. Bars represent mean±s.e.m., CTR (*n*=5) versus KO (*n*=9), ****P<0.0001 by analysis of variance (ANOVA) comparing the sustained phases. (**d**) Faeces sizes in CTR and *Tmem16a*^*SMKO*^ mice. *P<0.05; **P<0.01 by two-tailed Student's *t*-test, *n*=8 for each group. (**e**) A model of the molecular basis of basal tone generation in IAS. See text for the details. CaM/MLCK, camodulin/myosin light-chain kinase; p-RLC, phosphorylated 20-kDa myosin RLC; PM, plasma membrane; RyR, ryanodine receptor; SR, sarcoplasmic reticulum; VDCC, voltage-dependent calcium channel.

If a RyR-TMEM16A-Cav1 module is critical for the basal tone formation, genetic interruption of one of the members in this module could impair, or even abolish, the tone. Since IAS tissues express multiple RyRs and Cav1s, but only TMEM16A Cl_Ca_ channels, we tested this prediction by generating smooth-muscle-specific TMEM16A knockout mice. A conditional knockout of this gene is necessary because global deletion of TMEM16A causes post-natal lethality^36^, which makes force measurement in IAS quite a challenge. Supplementary Fig. 11a depicts the schematic of the strategy used to produce smooth-muscle-specific knockout mice with *Tmem16a* gene deletion, in which exon 12 was floxed with two loxP sites. That homologous recombination occurred in the floxed mice was confirmed with Southern blot analysis (Supplementary Fig. 11b). To delete TMEM16A specifically in smooth muscle, we crossed *Tmem16a* floxed mice with SMA-Cre transgenic mice^12^. The resultant mice *Tmem16a*^*flox/flox*^, *SMA-*Cre (that is, *Tmem16a*^*SMKO*^) were used as the KO mice, while *Tmem16a*^*flox/+*^, *SMA-Cre* littermates were used as control mice (CTR). Birth of *Tmem16a*^*SMKO*^ and CTR pups occurred in the expected Mendelian ratio. *Tmem16a*^*SMKO*^ mice were fertile, viable and lacked apparent developmental defects. Western blot demonstrated that TMEM16A in the KO IAS tissues was significantly decreased to ∼20% of the control levels (Supplementary Fig. 11c). The residual TMEM16A is most likely from other types of cells (for example, interstitial cells of Cajal (ICCs); Supplementary Fig. 12) in the IAS. To determine the changes in Cl_Ca_ currents due to TMEM16A deletion in IAS-SMCs, we compared these currents in cells from CTR and *Tmem16a*^*SMKO*^ mice. In the CTR cells, caffeine at 10 mM generated a Cl_Ca_ current of −1.67±0.37 pA pF^−1^ at the holding potential of −70 mV (*n*=12), while in the *Tmem16a*^*−/−*^ IAS-SMCs, the same concentration of caffeine yielded essentially no Cl_Ca_ current (−0.05±0.01 pA pF^−1^; *n*=14; Fig. 5a). To further characterize Cl_Ca_ currents in these cells, the IAS cells from both the KO and CTR were dialyzed with 600 nM [Ca^2+^]_i_ via the patch pipette. Out of 29 control cells, 6 cells produced a current of 36.54±6.18 pA pF^−1^ (*n*=6) at +100 mV and showed an outward rectification and time-dependent activation at positive potentials (Supplementary Fig. 13); In the *Tmem16a*^*SMKO*^ cells, one cell out of 29 had about one-half that current (16.54 pA pF^−1^) at +100 mV, and the other 28 cells had markedly reduced currents with 6.31±0.30 pA pF^−1^ (*n*=28) at the same potential (Supplementary Fig. 13). These results confirm that *Tmem16a* encodes Cl_Ca_ channels in smooth muscle cells^34^,^37^,^38^,^39^,^40^. They further reveal that some of cytosolic factors that bind with TMEM16A (ref. 41) may be required to prevent TMEM16A from its rundown, a characteristic often observed in Cl_Ca_ currents from native smooth muscle cells and other cell types^42^,^43^.

We further examined potential functional changes in IAS as a result of TMEM16A deletion in IAS-SMCs. We first studied the effect of TMEM16A modulators on caffeine-induced Ca^2+^ signals in the *Tmem16a*^*SMKO*^ cells. We found that both 16A_inh-_A01 and E_act_ did not decrease caffeine-induced Ca^2+^ release, nor did nifedipine and FPL64176 affect caffeine-induced Ca^2+^ release (Fig. 5b). We next compared the basal tone of IAS in the CTR and *Tmem16a*^*SMKO*^ mice. As shown in Fig. 5c, TMEM16A deficient IAS tissue produced ∼50% of the tone compared with the CTR IAS. We finally determined whether TMEM16A deletion in IAS-SMCs affect faecal continence by comparing the size of faeces from CTR and *Tmem16a*^*SMKO*^ mice (Fig. 5d). We found that faeces from *Tmem16a*^*SMKO*^ were both longer and wider (length: 5.4±0.26 mm in CTR versus 7.2±0.3 mm in *Tmem16a*^*SMKO*^; *n*=7 for each group, *P*<0.01 by two-tailed Student's *t*-test; width: 1.9±0.1 mm in CTR versus 2.3±0.1 mm in *Tmem16a*^*SMKO*^, *n*=7 for each group, *P*<0.05 by the *t*-test). These results indicate that TMEM16A is required for Cl_Ca_ currents in IAS-SMCs, and its deficiency impairs IAS basal tone formation and faecal continence.

## Discussion

Our results support a model in which RyRs, TMEM16A Cl_Ca_ channels and L-type VDCCs in IAS-SMCs form a signalling module to regulate global [Ca^2+^]_i_, which activates MLCK and sets IAS basal tone (Fig. 5e). This model is supported by several lines of evidence. First, genetic deletion of MLCK or TMEM16A specifically in IAS-SMCs abolishes or severely impairs the basal tone, leading to faecal impairment. Second, pharmacological activation of RyRs, TMEM16A or L-type Ca^2+^ channels increases the tone, while their blockage suppresses it. Third, the RyR-mediated increase in global [Ca^2+^]_i_ induced by caffeine is enhanced by TMEM16A agonist E_act_ and L-type Ca^2+^ channel agonist FPL64176, and inhibited by TMEM16A antagonist 16A_inh-_A01 and L-type Ca^2+^ channel blocker nifedipine. Fourth, the aforementioned effects by the modulators of TMEM16A and L-type Ca^2+^ channels are abolished when TMEM16A in IAS-SMCs is deleted. Finally, a global increase in [Ca^2+^]_i_ activates Cl_Ca_ channels, as directly recorded by patch clamp. Our model provides a molecular explanation for a long-standing notion that the basal tone in IAS is intrinsic to smooth muscle and independent of external stimuli^1^,^2^.

In our model, calcium release from the opening of RyRs in the sarcoplasmic reticulum of IAS-SMCs could be an initial signal for the basal tone generation and maintenance. The increased calcium then activates TMEM16A Cl_Ca_ channels subsequently activating L-type Ca^2+^ channels, resulting in Ca^2+^ influx. Given that TMEM16A has a low-Ca^2+^ sensitivity with an EC_50_ at ∼3 μM at −70 mV (refs 31, 44), an unexpected finding is that although Ca^2+^ sparks, a phenotypical localized Ca^2+^ release event, are present in IAS-SMCs, they do not activate TMEM16A Cl_Ca_ channels to generate STICs as in other smooth muscle cells^26^. Instead, a global rise in [Ca^2+^]_i_ created by activating RyRs is required to activate TMEM16A Cl_Ca_ channels, which in turn depolarizes the membrane and activates VDCCs, leading to the Ca^2+^-MLCK signalling cascade. Our results motivate three new questions. The first regards the reasons for the lack of STICs in IAS-SMCs. One possibility is that TMEM16A Cl_Ca_ channels do not concentrate near enough to Ca^2+^ spark sites. Since the unitary conductance of TMEM16A Cl_Ca_ channels is on the order of a few picosiemens, it requires ∼300 of them to localize near a spark site in order for a STIC to be generated. A direct visualization of TMEM16A Cl_Ca_ channels and RyRs at high spatial resolution will help solve this puzzle. The second question concerns the endogenous signalling molecules that convert local calcium events like Ca^2+^ sparks to a global rise in [Ca^2+^]_i_. One possibility is that applied stretch may activate a diffusible messenger such as cyclic ADP-ribose (cADPR), a potential endogenous ligand for RyR, or a stretch-gated ion channel. In this regard, it is worth mentioning that stretch can activate the non-selective cation channel TRPV4, which is known to activate Ca^2+^ sparks in vascular smooth muscle^45^. And the third question raised is the mechanism which terminates RyR-TMEM16A-L-type VDCC signalling. Interestingly, in smooth muscle Ca^2+^ levels in both the cytosol and the internal Ca^2+^ store can auto-regulate RyR activity^28^,^46^,^47^, and high Ca^2+^ in the cytosol inhibits L-type VDCCs and Cl_Ca_ channels^48^,^49^. One or more of these regulators may be able to terminate the RyR-TMEM16A-L-type VDCCs signalling process in IAS-SMCs. The activation and termination of this signalling module also imply that global [Ca^2+^]_i_ in these cells could be oscillating during basal tone development and maintenance. It would be interesting to examine whether an oscillating [Ca^2+^]_i_ leads to a low level of RLC phosphorylation during tone generation as revealed in Fig. 1e,f.

Out of two identified Cl_Ca_ channel genes^32^,^35^, we detected the expression of TMEM16A but not TMEM16B in IAS, consistent with the report that TMEM16B is predominantly expressed in the nervous system^35^. Interestingly, TMEM16A is thought to be absent from smooth muscle cells in the gastrointestinal tract where they generate phasic contractions^30^. With evidence from electrophysiological recordings, functional assays, RT–PCR and immunofluorescence, we have unambiguously demonstrated that TMEM16A is present and functional in IAS-SMCs. More importantly, using TMEM16A smooth muscle conditional knockout mice, we established that this gene is required for the generation and maintenance of basal tone in IAS. The fact that TMEM16A is expressed in sphincter smooth muscle cells while it is absent in phasic GI smooth muscle cells suggests this gene may be a differentiator for the phenotype of sphincters and phasic muscles. This finding suggests that TMEM16A may be an attractive therapeutic target for IAS motility disorders.

In the TMEM16A deficient mice, the tone was decreased by 51% compared with littermate controls. What accounts for this remaining tone? One possibility is that Cre in a small subset of IAS-SMCs has an insufficient efficiency, resulting in an incomplete deletion of the TMEM16A in these cells. Another possibility could be that activation of RyRs alone (that is, without amplification due to TMEM16A and L-type VDCCs) is sufficient to generate a certain amount of IAS tone. This is supported by our finding that caffeine-induced Ca^2+^ release is intact in IAS-SMCs from TMEM16A knockout mice (Fig. 5b). Finally the remaining tone could be attributed to the TMEM16A in ICCs in IAS tissues (Supplementary Fig. 12). In some types of smooth muscle a depolarization in ICCs can propagate to smooth muscle cells via gap junctions^50^. Whether this is the case in IAS-ICCs is not clear as IAS tone and slow waves are similar in control and *W/W*^*V*^ mice (that is, ICC depleted mice)^51^,^52^. Nevertheless, the presence of TMEM16A in the ICCs in IAS would require the application of an ICC cell specific knockout technique to uncover the role of ICC's TMEM16A in IAS function. Our study shows the power of this technique in addressing the role of TMEM16A in the IAS smooth muscle and so did by Heinze *et al.* 2014 who also elegantly demonstrated its role in the blood vessel smooth muscle^38^.

It is interesting that TMEM16A unevenly distributes across IAS smooth muscle, with the highest concentration apparently in the region adjacent to the submucosal space. This pattern of distribution implies that mechanical stretch by faeces may be able to preferentially activate TMEM16A in IAS smooth muscle, making this channel an attractive sensor of anal contents. This is in line with findings that membrane stretch can activate TMEM16A in vascular smooth muscle cells^40^, perhaps through the actin cytoskeleton^53^. This role for TMEM16A necessitates gap junctions among IAS-SMCs to propagate the electrical signals initiated in Cl_Ca_ channels near the submucosal space to the entire tissue. This is certainly possible as gap junctions are expressed abundantly in IAS cells (Supplementary Fig. 14) and other smooth muscle sphincter tissue^54^,^55^.

In summary, with an integrative approach combining genetically modified mice, bioassays, molecular biology and electrophysiology, we have uncovered the molecular mechanism underlying the genesis of spontaneous basal tone in IAS. This understanding will facilitate further insight into the pathophysiology of IAS disorders and the therapeutic options for treating them, as well as many other diseases related to smooth muscle sphincters.

## Methods

### Preparation of IAS tissue

C57BL/6, MYPT1 CTR (Mypt1^flox/+^; SMA-Cre) and Mypt1^SMKO^ (Mypt1^flox/flox^; SMA-Cre) mice, TMEM16A CTR (Tmem16a^flox/+^; SMA-Cre) and Tmem16a^SMKO^ (Tmem16a^flox/flox^; SMA-Cre) mice at 6–10 weeks were decapitated. MLCK CTR (Mlck^flox/+^; SM22-Cre^ERT2^) and Mlck^SMKO^ (Mlck^flox/flox^; SM22-Cre^ERT2^) mice were decapitated 20 days after tamoxifen injection. MYPT1 CTR, Mypt1^SMKO^, MLCK CTR and Mlck^SMKO^ mice were in a C57BL/6 background. TMEM16A CTR and Tmem16a^SMKO^ mutant mice were in a mixed C57BL/6 and Sv/129 background. Both genders of the mice were used equally. The anal canal and an adjacent region of the rectum were quickly removed and transferred to ice-cold and oxygenated Krebs physiological buffer (KPS) which was comprised of (in mM): 118.07 NaCl, 4.69 KCl, 2.52 CaCl_2_, 1.16 MgSO_4_, 1.01 NaH_2_PO_4_, 25 NaHCO_3_ and 11.10 glucose. Strained skeletal muscle fibres, mucosal layer and other extraneous tissues (for example, large blood vessels) were carefully dissected away, whereas the anal canal was left intact. The IAS was identified as a thickened circular smooth muscle situated at the lowermost part of anal canal, and the strips of IAS smooth muscle (∼1 × 6 mm) were prepared for experiments.

Animal experiments in this study were conducted in accordance with the guidelines of the Animal Care and Use Committee of Model Animal Research Center of Nanjing University (Nanjing, China), or University of Massachusetts Medical School, Massachusetts, USA.

### Measurement of IAS basal tone and contractility

Isolated strips of IAS smooth muscle were transferred to 8 ml muscle baths containing ice-cold oxygenated KPS. One end of the smooth-muscle strip was anchored to the bottom of the muscle bath. The other end of the smooth-muscle strip was connected to a force transducer (MLT0202, AD Instruments) and isometric tension was measured by a PowerLab (AD Instruments) recording device. A wire myograph (610-M, Danish Myo Technology, Aarhus, Denmark) was also used for force measurements. Each smooth-muscle strip was equilibrated for 60 min followed by a 0.5-g load^8^,^9^. The basal tone measurement is described in Supplementary Fig. 1. For evoked contractility measurements (that is, Supplementary Fig. 3), the resting tension of the IAS was adjusted to ∼0.2 g after developing spontaneous tone. KPS containing (in mM) 124 KCl, 2.52 CaCl_2_, 1.16 MgSO_4_, 1.01 NaH_2_PO_4_, 25 NaHCO_3_ and 11.10 glucose was used to achieve membrane depolarization. Bethanechol (100 μM) was used to induce agonist-induced contraction in IAS. For RyR inhibitor ryanodine experiments, IAS strips were pretreated with 100 μM ryanodine for 5 min before giving the initial stretch. For RyR agonist caffeine experiments, 3 mM caffeine was added into the bath 8 min after the initial stretch. Both ryanodine and caffeine were present through the experiments after administration. For L-type VDCC modulators (nifedipine and FPL64176) and Cl_Ca_ channel modulators (E_act_, niflumic acid or 16A_inh_-A01), experiments were performed in the presence of 1 μM atropine and 10 μM guanethidine to eliminate the possible effects of cholinergic and sympathetic nerves on the basal tone.

### Generation of *Tmem16a* knockout mice (*Tmem16a* ^*SMKO*^)

Bacterial artificial chromosome retrieval methods were used for constructing the TMEM16A targeting vector^56^. Briefly, the *Tmem16a* locus including Exon 12 was retrieved from a 129/sv bacterial artificial chromosome clone (bMQ 379h21, provided by the Sanger Institute) by a retrieval vector containing two homologous arms. Exon 12 which encodes a partial transcript in the second trans-member domain was floxed by 2 loxP sites, and an frt-Neo-frt cassette was inserted as a positive selection marker (Supplementary Fig. 11a). The deletion of this domain causes an out-of-frame reading shift and thereby generates a premature stop codon and a loss-of-function allele^36^. Embryonic stem W4 cells were electroporated with the linearized targeting vector, selected by long-fragment PCR and Southern blot analysis. Chimeric mice were generated by injecting homologous recombined embryonic stem cells into the blastocysts of C57BL/6 mice. Floxed *Tmem16a* mice with germ-line transmission were further confirmed by genotyping analysis and Southern blot analysis. To generate smooth-muscle-specific *Tmem16a* knockout mice, floxed *Tmem16a* mice were crossed with SMA-Cre mice.

### Isolation of mouse IAS-SMCs

Mice as listed above from 6 to 10 weeks of age of both genders were decapitated or euthanized with CO_2_. IAS was quickly removed and placed in a pre-chilled dissociation solution consisting of (in mM): 135 NaCl, 6 KCl, 5 MgCl_2_, 0.1 CaCl_2_, 0.2 EDTA, 10 Hepes and 10 glucose (pH 7.3). The tissue was then incubated in the dissociation medium containing 30 unit per ml papain, 1 mM DTT and 0.5 mg ml^−1^ BSA, at 35 °C for 30 min. The tissue was then transferred to a dissociation medium containing 3 unit per ml collagenase F and 0.5 mg ml^−1^ BSA, and incubated at 35 °C for another 15 min to produce isolated IAS cells. Finally, the tissue was agitated with a fire polished wide-bore glass pipette to release the cells.

### Western blot assay

IAS smooth muscle strips were homogenated in 120 μl lysis buffer (containing 20 mM Tris base, 137 mM NaCl, 2 mM EDTA, 1% NP-40, 10% glycerol, protease inhibitor cocktail (Roche), pH=8). Then the homogenization was incubated on ice for 15 min, and centrifugation at 3,000*g*, 4 °C to remove cell debris. Protein concentration was measured with a bicinchoinic acid protein assay kit (Pierce). The proteins were denatured at 95 °C for 5 min with sample buffer and reducing agent (5 × sample buffer contains 10% SDS, 20% glycerol, 0.05% bromophenol blue 10 mM β-mercapto-ethanol, 200 mM Tris-HCl and 8M urea). General SDS-page processes were followed. The blots were visualized by using the enhanced chemiluminescence method, the Super Signal West Dura substrate (PIERCE) and MaxiSignal Western Solution (SUDGEN) were used. All the primary antibodies used in western blot assay are listed in Supplementary Table 1, and the full scans of western blots are available in Supplementary Figs 15–17.

### Immunohistochemical analyses

Anal tubes were isolated and fixed in ice-cold acetone for 10 min, then washed with PBS overnight at 4 °C and rewashed for 4 h with a change of PBS per hour. Cryosections with a 10-μm thickness were used. The non-specific binding of primary antibodies was blocked by incubation with PBS containing 1% BSA and 5% non-immune goat serum for 1 h. Incubation was carried out overnight at 4 °C with a rabbit polyclonal antibody to TMEM16A (ab53212, abcam) diluted 1:200, together with a rat anti-c-Kit antibody (ACK2, Chemicon, 1:100) or mouse monoclonal Myh11 antibody (ab683 clone 1G12, 1:200; abcam). After washing in PBST, sections were incubated with a Alexa Fluor 555-conjugated goat anti-Rabbit antibody (Sigma) diluted 1:250 and a FITC-conjugated goat anti-rat antibody (Invitrogen) diluted 1:250 or a Alexa Fluor 488–conjugated goat anti-mouse antibody (Cell signaling technology) for 1 h. 4,6-diamidino-2-phenylindole was used for nuclear staining. Immunoreactivity was evaluated using a FV1000 confocal laser scanning microscope system (Olympus).

### Patch-clamp recording

Membrane currents were recorded using the perforated whole-cell patch recording configuration. The extracellular solution contained (in mM): 130 NaCl, 5.5 TEA-Cl, 2.2 CaCl_2_, 1 MgCl_2_, 10 Hepes and 5.6 glucose; pH adjusted to 7.4 with NaOH. The pipette solution contained (in mM): 137 CsCl, 1 MgCl_2_, 10 Hepes, 3 Na_2_ATP; pH adjusted to 7.3 with CsOH. Amphotericin B was freshly made and added to the pipette solution at a final concentration of 200 μg ml^−1^. Whole-cell currents were recorded when cells were held at the designated potentials and the currents were low-pass filtered using the Axopatch 1D amplifier (200 Hz cutoff) and then digitally sampled at 1 kHz and stored for analysis. Caffeine was applied to the cells via a puffing pipette placed ∼100 μm from the cells under the control of a picospritzer.

For recording of 600 nM Ca^2+^-induced currents, conventional whole-cell patch clamp was carried out. The extracellular solution contained (in mM) 144.5 NaCl, 5.5 TEA-Cl, 1 CaCl_2_, 1 MgCl_2_, 10 glucose, 10 mannitol and 10 Hepes; pH was adjusted to 7.4 with NaOH. The pipette solution contained (in mM) 130 CsCl, 10 EGTA, 1 MgCl_2_, 8 CaCl_2_, 10 Hepes, 1 MgATP; pH was adjusted to 7.3 with NaOH. Whole-cell currents were recorded in response to 1 s voltage pulses from −80 to +100 mV in 10 mV increments followed by 700 ms pulses to −60 mV, in freshly isolated IAS-SMCs. Holding potential was 0 mV. Currents were sampled at 20 kHz using the Axon MultiClamp 700B amplifier (Molecular Devices) and then low-pass filtered at 2 kHz.

For recording L-type VDCCs in IAS-SMCs, conventional whole-cell patch clamp was carried with extracellular solution containing (in mM) 130 TEA-Cl, 10 CaCl_2_, 1 MgCl_2_, 10 Hepes, 10 glucose; pH was adjusted to 7.4 with NaOH. The pipette solution contained (in mM) 130 CsCl, 4 MgCl_2_, 10 Hepes, 10 EGTA, 5 Na_2_ATP; pH was adjusted to 7.2 with CsOH. Cells were held at −80 mV, whole-cell currents were recorded in response to 200 ms voltage pulses from −80 to +50 mV in 10 mV increments. Freshly isolated IAS-SMCs were incubated in extracelluar solution with 1 μM Nifedipine or vehicle (0.1% EtOH) during recording. Currents were sampled at 20 kHz using the Axon MultiClamp 700B amplifier (Molecular Devices) and then low-pass filtered at 2 kHz.

### Imaging and measurement of Ca^2+^ sparks

Fluorescent images were obtained using fluo-3 as the Ca^2+^ indicator and a custom-built wide- field, high-speed digital imaging system^57^. Rapid imaging was made possible by using a cooled high-sensitivity charge-coupled device camera (128 × 128 pixels) developed in conjunction with the Massachusetts Institute of Technology Lincoln Laboratory. The camera was interfaced to a custom-made inverted microscope equipped with a × 60 oil immersion lens (numerical aperture of 1.3), with each pixel covering a 333 × 333-nm area of the cell. The 488-nm line of a multiline argon laser provided fluorescence excitation for the indicator fluo-3, and a laser shutter controlled the exposure duration. Emission of the Ca^2+^ indicator was monitored at wavelengths >510 nm. Ca^2+^ sparks were measured as the conventional fluorescence ratio (ΔF/F_0_) within a restricted volume as described previously^57^.

### Reverse transcription PCR detection of messenger RNA

The IAS and rectal smooth muscle (4 mm above IAS) from mice were carefully isolated, quickly removed from connective tissue and then frozen in liquid nitrogen. The total RNA was isolated using the TRIZOL (Invitrogen) method according to the manufacturer's guidelines, and cDNA was synthesized. Primers were synthesized by Invitrogen, and their sequences are available in Supplementary Table 2.

### Statistical analysis

Data are presented as the mean±s.e.m. Differences between groups were determined by Student's *t*-test, or two-way analysis of variance (ANOVA) for significant differences. The significance levels were indicated as follows: NS *P*>0.05, *P<0.05, **P<0.01, ***P<0.001, ****P<0.0001.

## Additional information

**How to cite this article:** Zhang, C.-H. *et al.* The molecular basis of the genesis of basal tone in internal anal sphincter. *Nat. Commun.* 7:11358 doi: 10.1038/ncomms11358 (2016).

## Supplementary Material

Supplementary Information Supplementary Figures 1-17, Supplementary Tables 1-2 and Supplementary References

## References

[b1] Vander A., Sherman J. & Luciano D. Human Physiology: The Mechanisms of Body Function 6th edn, International Edition McGraw-Hill international editions (1994) .

[b2] Rattan S. The internal anal sphincter: regulation of smooth muscle tone and relaxation. Neurogastroenterol. Motil. 17, 50–59 (2005) . 15836455 10.1111/j.1365-2982.2005.00659.x

[b3] Murthy K. S. Signaling for contraction and relaxation in smooth muscle of the gut. Annu. Rev. Physiol. 68, 345–374 (2006) . 16460276 10.1146/annurev.physiol.68.040504.094707

[b4] Somlyo A. P. & Somlyo A. V. Ca^2+^ sensitivity of smooth muscle and nonmuscle myosin II: modulated by G proteins, kinases, and myosin phosphatase. Physiol. Rev. 83, 1325–1358 (2003) . 14506307 10.1152/physrev.00023.2003

[b5] Rattan S. & Singh J. RhoA/ROCK pathway is the major molecular determinant of basal tone in intact human internal anal sphincter. Am. J. Physiol. Gastrointest. Liver Physiol. 302, G664–G675 (2012) . 22241857 10.1152/ajpgi.00430.2011 PMC3330775

[b6] Patel C. A. & Rattan S. Cellular regulation of basal tone in internal anal sphincter smooth muscle by RhoA/ROCK. Am. J. Physiol. Gastrointest. Liver Physiol. 292, G1747–G1756 (2007) . 17379756 10.1152/ajpgi.00438.2006

[b7] Patel C. A. & Rattan S. Spontaneously tonic smooth muscle has characteristically higher levels of RhoA/ROK compared with the phasic smooth muscle. Am. J. Physiol. Gastrointest. Liver Physiol. 291, G830–G837 (2006) . 16763289 10.1152/ajpgi.00130.2006

[b8] Mcdonnell B., Hamilton R., Fong M., Ward S. M. & Keef K. D. Functional evidence for purinergic inhibitory neuromuscular transmission in the mouse internal anal sphincter. Am. J. Physiol. Gastrointest. Liver Physiol. 294, G1041–G1051 (2008) . 18308858 10.1152/ajpgi.00356.2007

[b9] Chakder S., Mchugh K. M. & Rattan S. Inhibitory neurotransmission in lethal spotted mutant mice: a model for Hirschsprung's disease. Gastroenterology 112, 1575–1585 (1997) . 9136836 10.1016/s0016-5085(97)70039-8

[b10] Hall K. A., Ward S. M., Cobine C. A. & Keef K. D. Spatial organization and coordination of slow waves in the mouse anorectum. J. Physiol. 592, 3813–3829 (2014) . 24951622 10.1113/jphysiol.2014.272542 PMC4192705

[b11] Cobine C. A. *et al.* Interstitial cells of Cajal in the cynomolgus monkey rectoanal region and their relationship to sympathetic and nitrergic nerves. Am. J. Physiol. Gastrointest. Liver Physiol. 298, G643–G656 (2010) . 20150245 10.1152/ajpgi.00260.2009 PMC2867417

[b12] He W. Q. *et al.* Altered contractile phenotypes of intestinal smooth muscle in mice deficient in myosin phosphatase target subunit 1. Gastroenterology 144, 1456–1465 (2013) . 23499953 10.1053/j.gastro.2013.02.045 PMC3782749

[b13] He W. Q. *et al.* Myosin light chain kinase is central to smooth muscle contraction and required for gastrointestinal motility in mice. Gastroenterology 135, 610–620 (2008) . 18586037 10.1053/j.gastro.2008.05.032 PMC2648853

[b14] Zhang W. C. *et al.* Myosin light chain kinase is necessary for tonic airway smooth muscle contraction. J. Biol. Chem. 285, 17–19 (2010) . 10.1074/jbc.M109.062836 PMC2820780 20018858

[b15] Rao S., Kempf J. & Stessman M. Anal seepage: sphincter dysfunction or incomplete evaluation? Gastroenterology 114, A824 (1998) .

[b16] Rao S. S. Pathophysiology of adult fecal incontinence. Gastroenterology 126, S14–S22 (2004) . 14978634 10.1053/j.gastro.2003.10.013

[b17] Cobine C. A., Fong M., Hamilton R. & Keef K. D. Species dependent differences in the actions of sympathetic nerves and noradrenaline in the internal anal sphincter. Neurogastroenterol. Motil. 19, 937–945 (2007) . 17973631 10.1111/j.1365-2982.2007.00982.x

[b18] Zheng W., Rampe D. & Triggle D. J. Pharmacological, radioligand binding, and electrophysiological characteristics of FPL 64176, a novel nondihydropyridine Ca^2+^ channel activator, in cardiac and vascular preparations. Mol. Pharmacol. 40, 734–741 (1991) . 1719369

[b19] McDonough S. I., Mori Y. & Bean B. P. FPL 64176 modification of Ca_V_1.2L-type calcium channels: dissociation of effects on ionic current and gating current. Biophys. J. 88, 211–223 (2005) . 15501945 10.1529/biophysj.104.051714 PMC1304999

[b20] Zhuge R., Bao R., Fogarty K. E. & Lifshitz L. M. Ca^2+^ sparks act as potent regulators of excitation-contraction coupling in airway smooth muscle. J. Biol. Chem. 285, 2203–2210 (2010) . 19920135 10.1074/jbc.M109.067546 PMC2804376

[b21] Bulley S. & Jaggar J. H. Cl^−^ channels in smooth muscle cells. Pflug. Arch. 466, 861–872 (2013) . 10.1007/s00424-013-1357-2 PMC3969453 24077695

[b22] Saha J. K., Sengupta J. N. & Goyal R. K. Role of chloride ions in lower esophageal sphincter tone and relaxation. Am. J. Physiol. Gastrointest. Liver Physiol. 263, G115–G126 (1992) . 10.1152/ajpgi.1992.263.1.G115 1636707

[b23] Namkung W., Phuan P. W. & Verkman A. S. TMEM16A inhibitors reveal TMEM16A as a minor component of calcium-activated chloride channel conductance in airway and intestinal epithelial cells. J. Biol. Chem. 286, 2365–2374 (2011) . 21084298 10.1074/jbc.M110.175109 PMC3023530

[b24] White M. M. & Aylwin M. Niflumic and flufenamic acids are potent reversible blockers of Ca^2+^-activated Cl^-^ channels in Xenopus oocytes. Mol. Pharmacol. 37, 720–724 (1990) . 1692608

[b25] Namkung W., Yao Z., Finkbeiner W. E. & Verkman A. S. Small-molecule activators of TMEM16A, a calcium-activated chloride channel, stimulate epithelial chloride secretion and intestinal contraction. FASEB J. 25, 4048–4062 (2011) . 21836025 10.1096/fj.11-191627 PMC3205834

[b26] Nelson M. T. *et al.* Relaxation of arterial smooth muscle by calcium sparks. Science 270, 633–637 (1995) . 7570021 10.1126/science.270.5236.633

[b27] Zhuge R. *et al.* Ca^2+^ spark sites in smooth muscle cells are numerous and differ in number of ryanodine receptors, large-conductance K^+^ channels, and coupling ratio between them. Am. J. Physiol. Cell Physiol. 287, C1577–C1588 (2004) . 15306542 10.1152/ajpcell.00153.2004

[b28] Zhuge R. *et al.* The influence of sarcoplasmic reticulum Ca^2+^ concentration on Ca^2+^ sparks and spontaneous transient outward currents in single smooth muscle cells. J. Gen. Physiol. 113, 215–228 (1999) . 9925820 10.1085/jgp.113.2.215 PMC2223361

[b29] Zhuge R., Sims S. M., Tuft R. A., Fogarty K. E. & Walsh J. V. Jr Ca^2+^ sparks activate K^+^ and Cl^-^ channels, resulting in spontaneous transient currents in guinea-pig tracheal myocytes. J. Physiol. 513, 711–718 (1998) . 9824712 10.1111/j.1469-7793.1998.711ba.x PMC2231323

[b30] Huang F. *et al.* Studies on expression and function of the TMEM16A calcium-activated chloride channel. Proc. Natl Acad. Sci. USA 106, 21413–21418 (2009) . 19965375 10.1073/pnas.0911935106 PMC2781737

[b31] Yang Y. D. *et al.* TMEM16A confers receptor-activated calcium-dependent chloride conductance. Nature 455, 1210–1215 (2008) . 18724360 10.1038/nature07313

[b32] Schroeder B. C., Cheng T., Jan Y. N. & Jan L. Y. Expression cloning of TMEM16A as a calcium-activated chloride channel subunit. Cell 134, 1019–1029 (2008) . 18805094 10.1016/j.cell.2008.09.003 PMC2651354

[b33] Caputo A. *et al.* TMEM16A, a membrane protein associated with calcium-dependent chloride channel activity. Science 322, 590–594 (2008) . 18772398 10.1126/science.1163518

[b34] Manoury B., Tamuleviciute A. & Tammaro P. TMEM16A/anoctamin 1 protein mediates calcium-activated chloride currents in pulmonary arterial smooth muscle cells. J. Physiol. 588, 2305–2314 (2010) . 20421283 10.1113/jphysiol.2010.189506 PMC2915508

[b35] Stohr H. *et al.* TMEM16B, a novel protein with calcium-dependent chloride channel activity, associates with a presynaptic protein complex in photoreceptor terminals. J. Neurosci. 29, 6809–6818 (2009) . 19474308 10.1523/JNEUROSCI.5546-08.2009 PMC6665584

[b36] Rock J. R., Futtner C. R. & Harfe B. D. The transmembrane protein TMEM16A is required for normal development of the murine trachea. Dev. Biol. 321, 141–149 (2008) . 18585372 10.1016/j.ydbio.2008.06.009

[b37] Zhang C. H. *et al.* The transmembrane protein 16A Ca^2+^-activated Cl^-^ channel in airway smooth muscle contributes to airway hyperresponsiveness. Am. J. Respir. Crit. Care Med. 187, 374–381 (2013) . 23239156 10.1164/rccm.201207-1303OC PMC3603598

[b38] Heinze C. *et al.* Disruption of vascular Ca^2+^-activated chloride currents lowers blood pressure. J. Clin. Invest. 124, 675–686 (2014) . 24401273 10.1172/JCI70025 PMC3904609

[b39] Dam V. S., Boedtkjer D. M., Nyvad J., Aalkjaer C. & Matchkov V. TMEM16A knockdown abrogates two different Ca^2+^-activated Cl^−^ currents and contractility of smooth muscle in rat mesenteric small arteries. Pflug. Arch. 466, 1391–1409 (2014) . 10.1007/s00424-013-1382-1 PMC4062836 24162234

[b40] Bulley S. *et al.* TMEM16A/ANO1 channels contribute to the myogenic response in cerebral arteries. Circ. Res. 111, 1027–1036 (2012) . 22872152 10.1161/CIRCRESAHA.112.277145 PMC3666568

[b41] Perez-Cornejo P. *et al.* Anoctamin 1 (Tmem16A) Ca^2+^-activated chloride channel stoichiometrically interacts with an ezrin-radixin-moesin network. Proc. Natl Acad. Sci. USA 109, 10376–10381 (2012) . 22685202 10.1073/pnas.1200174109 PMC3387097

[b42] Angermann J. E., Sanguinetti A. R., Kenyon J. L., Leblanc N. & Greenwood I. A. Mechanism of the inhibition of Ca^2+^-activated Cl^-^ currents by phosphorylation in pulmonary arterial smooth muscle cells. J. Gen. Physiol. 128, 73–87 (2006) . 16801382 10.1085/jgp.200609507 PMC2151553

[b43] Morris A. P. & Frizzell R. A. Ca^2+^-dependent Cl^-^ channels in undifferentiated human colonic cells (HT-29). II. Regulation and rundown. Am. J. Physiol. Cell Physiol. 264, C977–C985 (1993) . 10.1152/ajpcell.1993.264.4.C977 7682780

[b44] Bao R. *et al.* A close association of RyRs with highly dense clusters of Ca^2+^-activated Cl^-^ channels underlies the activation of STICs by Ca^2+^ sparks in mouse airway smooth muscle. J. Gen. Physiol. 132, 145–160 (2008) . 18591421 10.1085/jgp.200709933 PMC2442178

[b45] Morita H. *et al.* Membrane stretch-induced activation of a TRPM4-like nonselective cation channel in cerebral artery myocytes. J. Pharmacol. Sci. 103, 417–426 (2007) . 17420615 10.1254/jphs.fp0061332

[b46] Collier M., Ji G., Wang Y. X. & Kotlikoff M. Calcium-induced calcium release in smooth muscle loose coupling between the action potential and calcium release. J. Gen. Physiol. 115, 653–662 (2000) . 10779321 10.1085/jgp.115.5.653 PMC2217224

[b47] Essin K. *et al.* Indirect coupling between Ca_V_1. 2 channels and ryanodine receptors to generate Ca^2+^ sparks in murine arterial smooth muscle cells. J. Physiol. 584, 205–219 (2007) . 17673505 10.1113/jphysiol.2007.138982 PMC2277062

[b48] Carl A., Lee H. K. & Sanders K. M. Regulation of ion channels in smooth muscles by calcium. Am. J. Physiol. Cell Physiol. 271, C9–C34 (1996) . 10.1152/ajpcell.1996.271.1.C9 8760027

[b49] Piper A. S. & Large W. A. Multiple conductance states of single Ca^2+^-activated Cl^-^ channels in rabbit pulmonary artery smooth muscle cells. J. Physiol. 547, 181–196 (2003) . 12562904 10.1113/jphysiol.2002.033688 PMC2342635

[b50] Sanders K. M., Ward S. M. & Koh S. D. Interstitial cells: regulators of smooth muscle function. Physiol. Rev. 94, 859–907 (2014) . 24987007 10.1152/physrev.00037.2013 PMC4152167

[b51] Duffy A. M., Cobine C. A. & Keef K. D. Changes in neuromuscular transmission in the W/W^V^ mouse internal anal sphincter. Neurogastroenterol. Motil. 24, e41–e55 (2012) . 22074497 10.1111/j.1365-2982.2011.01806.x PMC3245326

[b52] de Lorijn F. *et al.* Interstitial cells of Cajal are involved in the afferent limb of the rectoanal inhibitory reflex. Gut 54, 1107–1113 (2005) . 16009682 10.1136/gut.2004.051045 PMC1774907

[b53] Ohshiro J., Yamamura H., Suzuki Y. & Imaizumi Y. Modulation of TMEM16A-channel activity as Ca^2+^ activated Cl^-^ conductance via the interaction with actin cytoskeleton in murine portal vein. J. Pharmacol. Sci. 125, 107–111 (2014) . 24770592 10.1254/jphs.14015sc

[b54] Wang Y. F. & Daniel E. E. Gap junctions in gastrointestinal muscle contain multiple connexins. Am. J. Physiol. Gastrointest. Liver Physiol. 281, G533–G543 (2001) . 11447034 10.1152/ajpgi.2001.281.2.G533

[b55] Samuel U., Lutjen-Drecoll E. & Tamm E. R. Gap junctions are found between iris sphincter smooth muscle cells but not in the ciliary muscle of human and monkey eyes. Exp. Eye Res. 63, 187–192 (1996) . 8983976 10.1006/exer.1996.0107

[b56] Liu P., Jenkins N. A. & Copeland N. G. A highly efficient recombineering-based method for generating conditional knockout mutations. Genome Res. 13, 476–484 (2003) . 12618378 10.1101/gr.749203 PMC430283

[b57] ZhuGe R. *et al.* Dynamics of signaling between Ca^2+^ sparks and Ca^2+^- activated K^+^ channels studied with a novel image-based method for direct intracellular measurement of ryanodine receptor Ca^2+^ current. J. Gen. Physiol. 116, 845–864 (2000) . 11099351 10.1085/jgp.116.6.845 PMC2231814

