# Supplementary material for: The molecular basis of the genesis of basal tone in internal anal sphincter
Source: Nat Commun. 2016 Apr 22;7:11358. doi: 10.1038/ncomms11358 (PMC4844698; doi:10.1038/ncomms11358)
Supplement: Supplementary Information — Supplementary Figures 1-17, Supplementary Tables 1-2 and Supplementary References [file ncomms11358-s1.pdf]

Supplementary Information

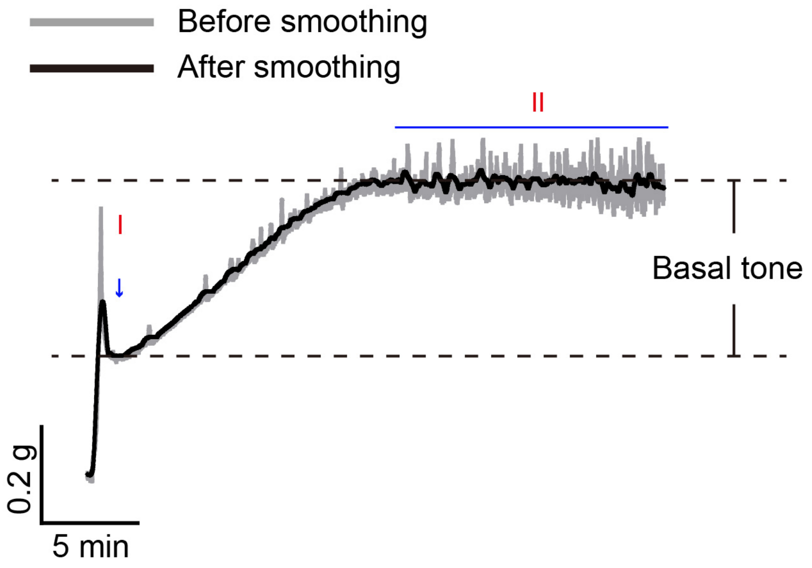

Supplementary Figure 1. Basal tone quantification. The basal tone was defined as the tension difference between the lowest value (i.e., "I") after the load was applied and the value when the tone reached a plateau (i.e., "II") after the load. Since occasionally IAS tissues generate phasic contractions superimposed on the basal tone (e.g., this figure and Supplementary Fig. 6), a 30 second (600 points at 20 Hz sampling frequency) smoothing was applied to the original force traces so the lowest value and the sustained value could be measured accurately.

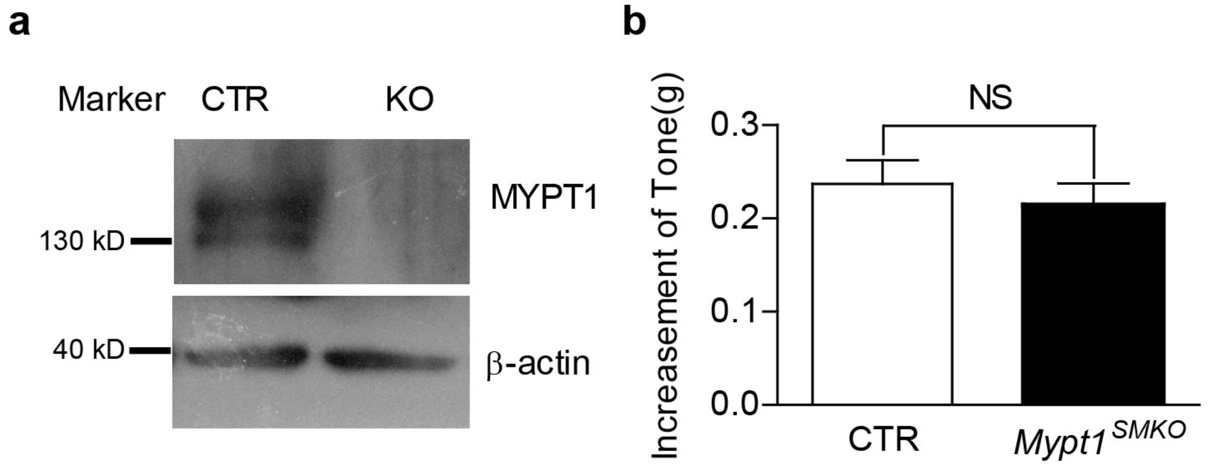

Supplementary Figure 2. MYPT1 deletion exerts no effect on the amplitude of the IAS basal tone in mice. (a) Western blots of MYPT1 in IAS from control mice and MYPT1 deficient mice. MYPT1 expression is totally deleted in *Mypt1*^{SMKO} mice. β-actin was used as a protein loading control. (b) Quantification of basal tone in control IAS and MYPT1 deficient IAS. Bars represent mean ± s.e.m., n=13 for CTR and n=15 for *Mypt1*^{SMKO} NS: no significant difference between control and knockout group by two tailed student's *t*-test.

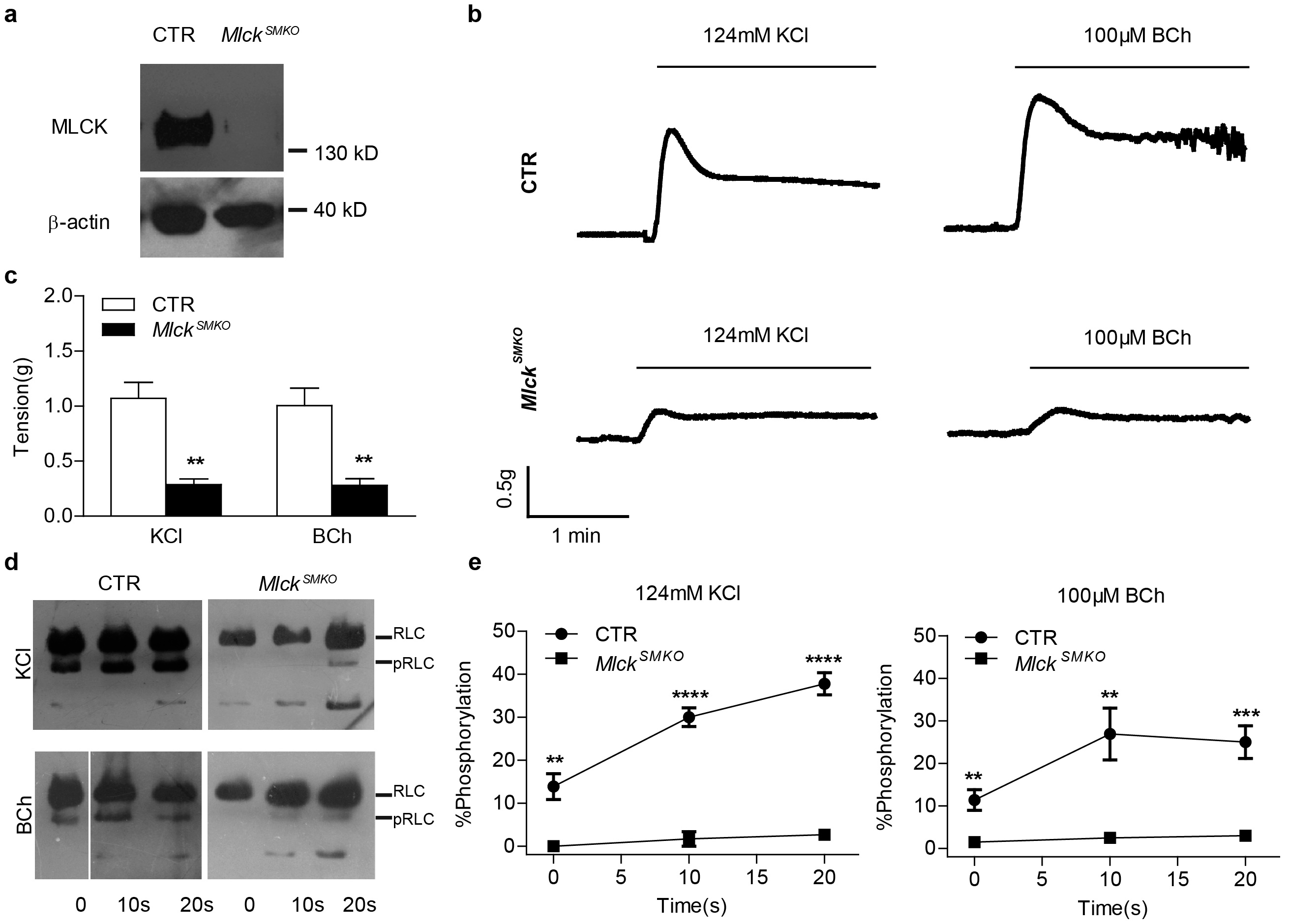

Supplementary Figure 3. Impaired agonist-induced and depolarization-induced contractions and reduced RLC phosphorylation in the IAS from *Mlck*^{SMKO} mice. (a) Western blots of MLCK in control and *Mlck*^{SMKO} IAS collected 20 days after tamoxifen injection. β-actin was used as the protein loading control. (b) Representative recordings of IAS contraction in response to 124 mM KCl or 100 μM bethanechol (BCh) in CTR mice and *Mlck*^{SMKO} mice. (c) Quantification of contractile responses (peak tension) in response to 124 mM KCl and 100 μM BCh. Bars represent mean ± s.e.m., n=5–7, $**P < 0.01$ by two-tailed student's *t*-test. (d) and (e) The changes in RLC phosphorylation (RLCp) as assessed with western blots of glycerol/urea PAGE gel in quickly frozen IAS tissues after treated with 124 mM KCl or 100 μM BCh. Bars represent mean ± s.e.m., n=4–10, $**P< 0.01$, $***P<0.001$, $****P<0.0001$ by two-tailed student's *t*-test.

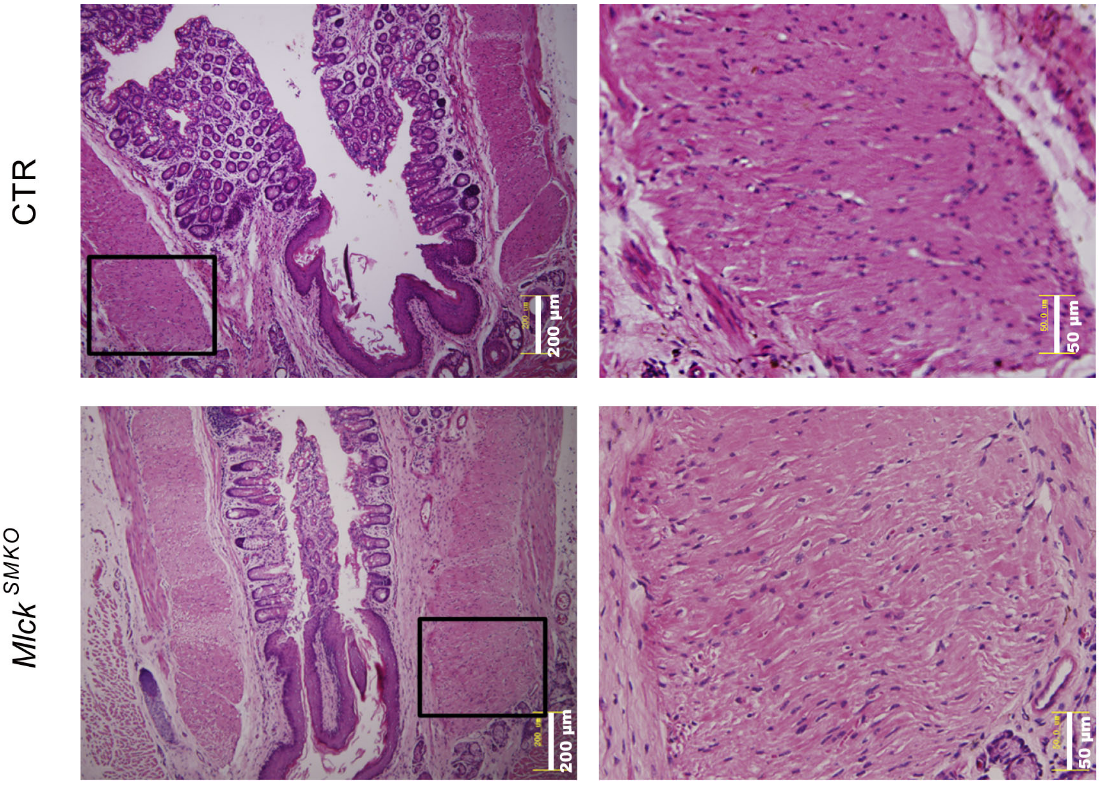

Supplementary Figure 4. Histological analyses of IAS from CTR and *Mlck*^{SMKO} mice. Left panels are images of the entire rectoanal region (scale bars: 200 µm) and right panels show IAS (scale bar: 50 µm) as marked by the boxes in the left images.

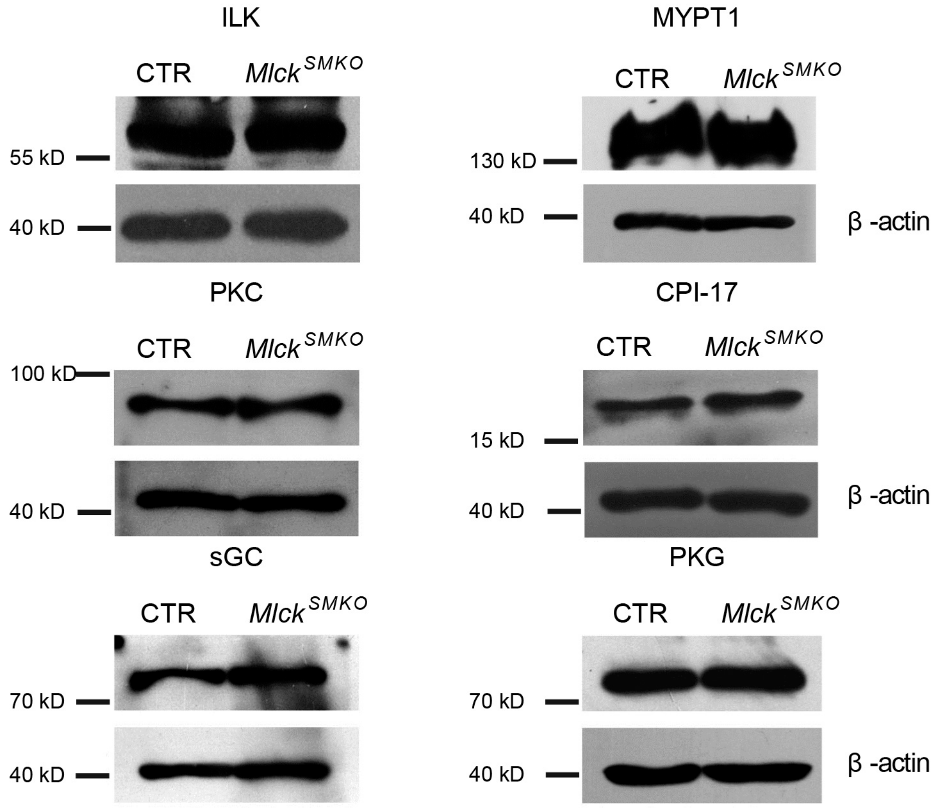

Supplementary Figure 5. Contractile and related regulatory proteins are the same in the IAS from control and MLCK-deficient mice. Samples were resolved by separate SDS-PAGE, and β-actin was stained as a loading control. Blots are representative of at least three measurements.

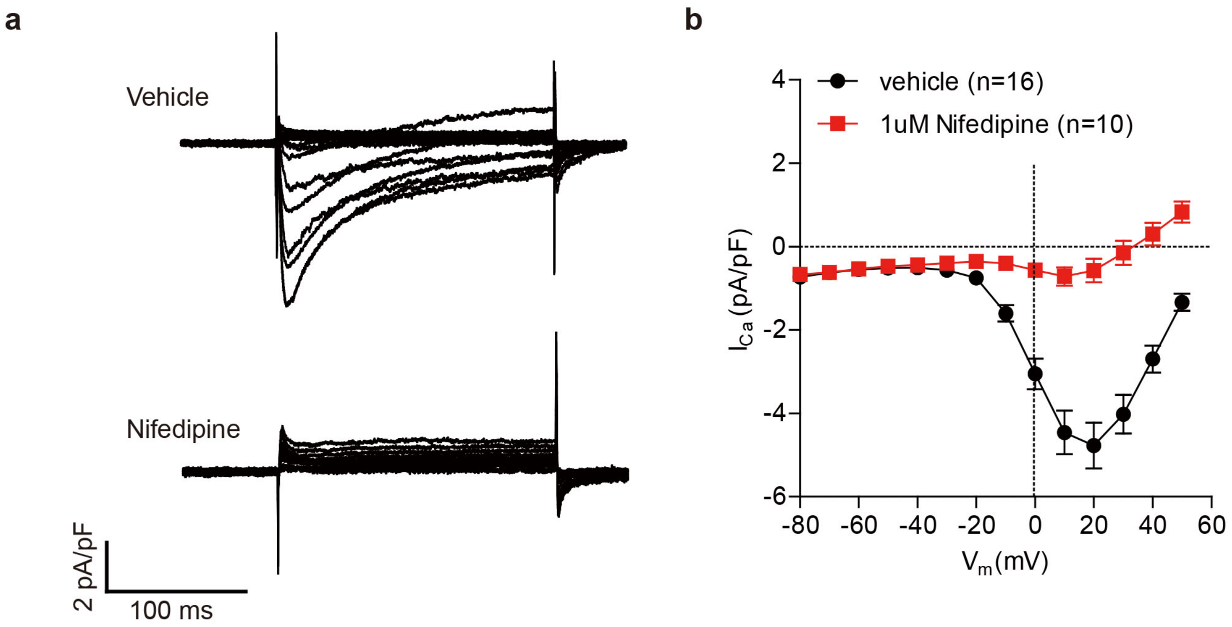

Supplementary Figure 6. Mouse IAS smooth muscle cells express functional L-type VDCCs. (a) Two original recordings showing typical L-type Ca^{2+} currents in response to a train of voltage depolarization (upper panel) and their blockage by 1µM nifedipine (lower panel). (b) The I-V curves of L-type currents and their blockage by nifedipine as determined in the experiments in a.

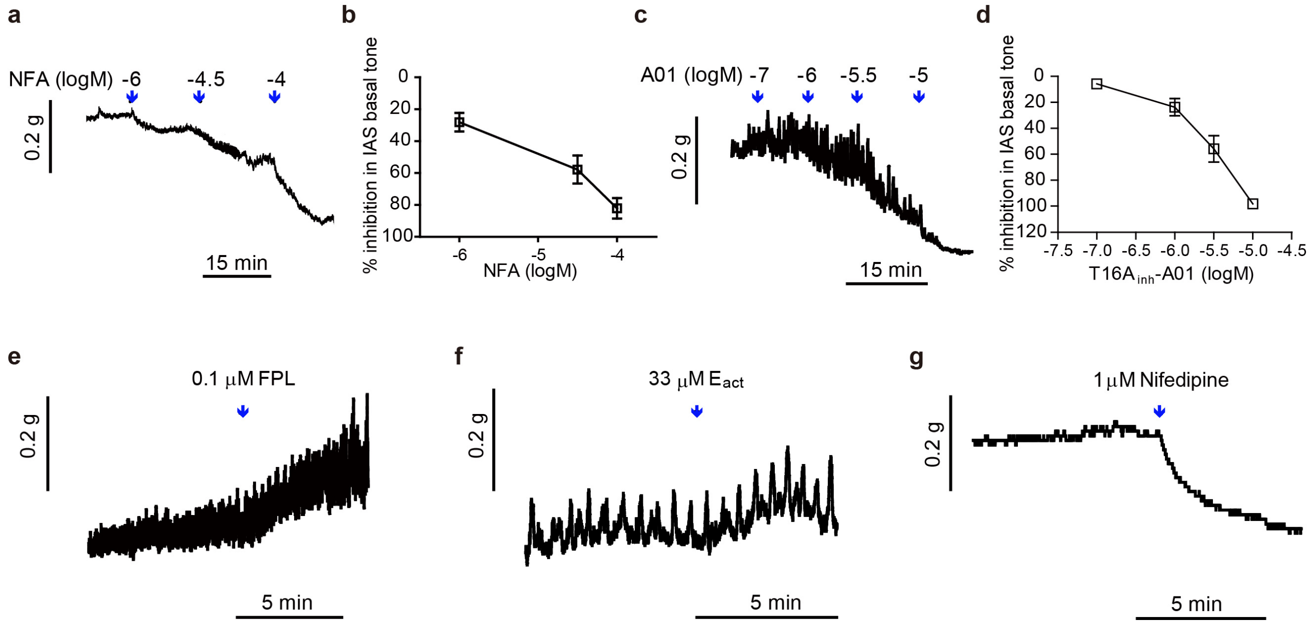

Supplementary Figure 7. Effects of Cl_{Ca} channel and L-type VDCC modulators on mouse IAS basal tone. (a) & (b) A typical trace of Cl_{Ca} channel inhibitor niflumic acid-induced relaxation of IAS basal tone at different concentrations and its quantitative data curve. Bars represent mean ± s.e.m., n=4–8. (c) & (d) A typical trace of TMEM16A Cl_{Ca} channel inhibitor $16A_{inh}$-A01-induced relaxation of IAS basal tone at different concentrations and its quantitative data curve. Bars represent mean ± s.e.m., n=4. (e) A typical trace of L-type VDCC agonist FPL-induced potentiation of IAS basal tone. (f) A typical trace of Tmem16A Cl_{Ca} channel agonist E_{act}-induced potentiation of IAS basal tone. (g) A typical trace of L-type VDCC blocker nifedipine-induced relaxation of IAS basal tone.

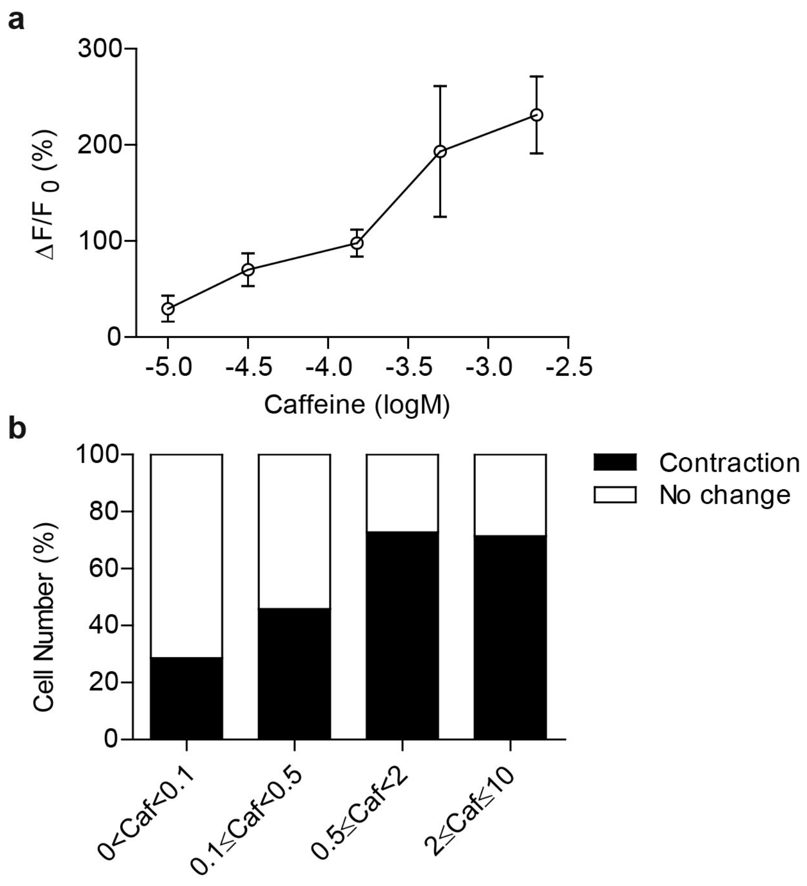

Supplementary Figure 8. IAS is highly sensitive to caffeine. (a) Mean dose response of a caffeine-induced Ca^{2+} increase in isolated IAS SMCs. Note this response is more sensitive than in other smooth muscle cells[1]. Bars represent mean ± s.e.m.. (b) Percentage of contracted and non-contracted IAS smooth muscle cells following stimulation with caffeine (Caf) at different concentrations, n=46.

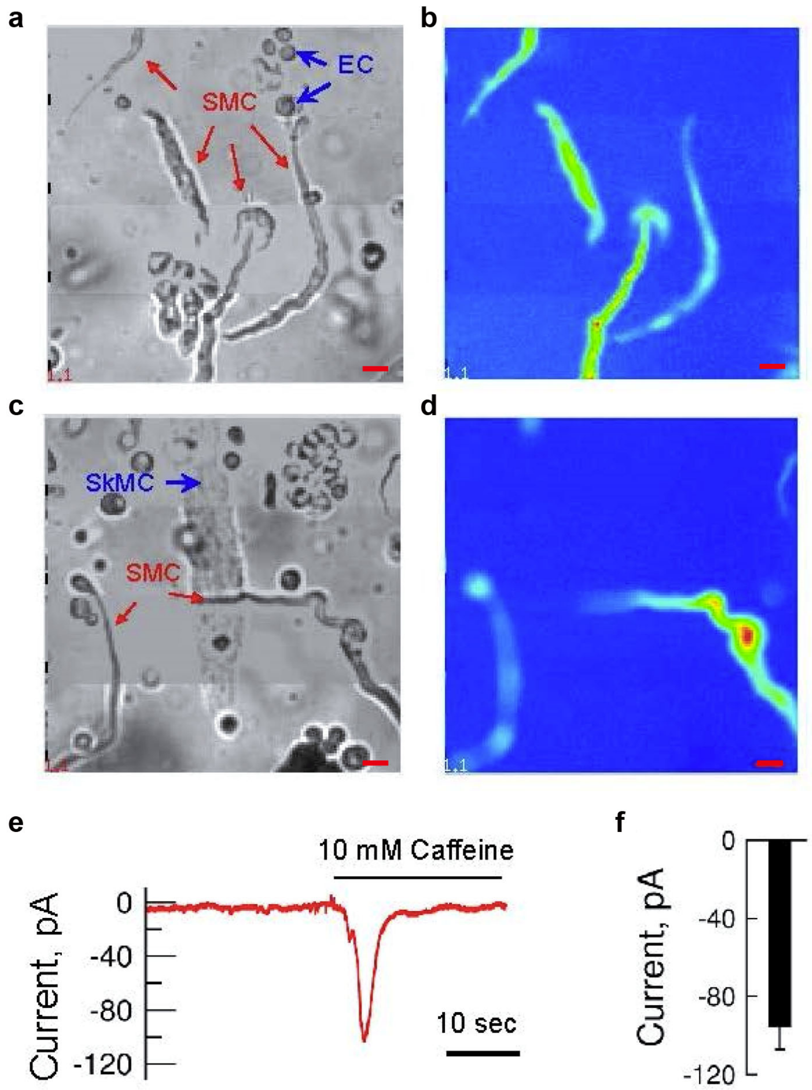

Supplementary Figure 9. IAS smooth muscle cells (SMCs) from α-smooth muscle actin (αSMA)-GFP mice generate caffeine-induced Ca^{2+}-activated Cl^- currents. IAS cells were isolated from αSMA-hrGFP mice (a gift from Dr. Alan Fine at Boston University) where hrGFP is expressed under the control of αSMA gene promoter[2]. (a) & (b) are the same cells illuminated with white light and a 488 nm laser, respectively. SMCs (marked by red arrows) contain GFP while epithelial cells (EC, two of them are marked by blue arrows) have no GFP. Red scale bars in panels a, b and other panels in this figure equal 10 µm. (c) & (d) were imaged in the same way as in (a) and (b). SMCs (marked by red arrows) contain GFP while a skeletal muscle cell (SkMC) shows no GFP. (e) An example of caffeine-induced Ca^{2+}-activated Cl^- current in a GFP positive IAS SMC held at -70 mV under perforated patch clamp. Panel (f) is the mean ± s.e.m. (n=3) from the experiments in (e).

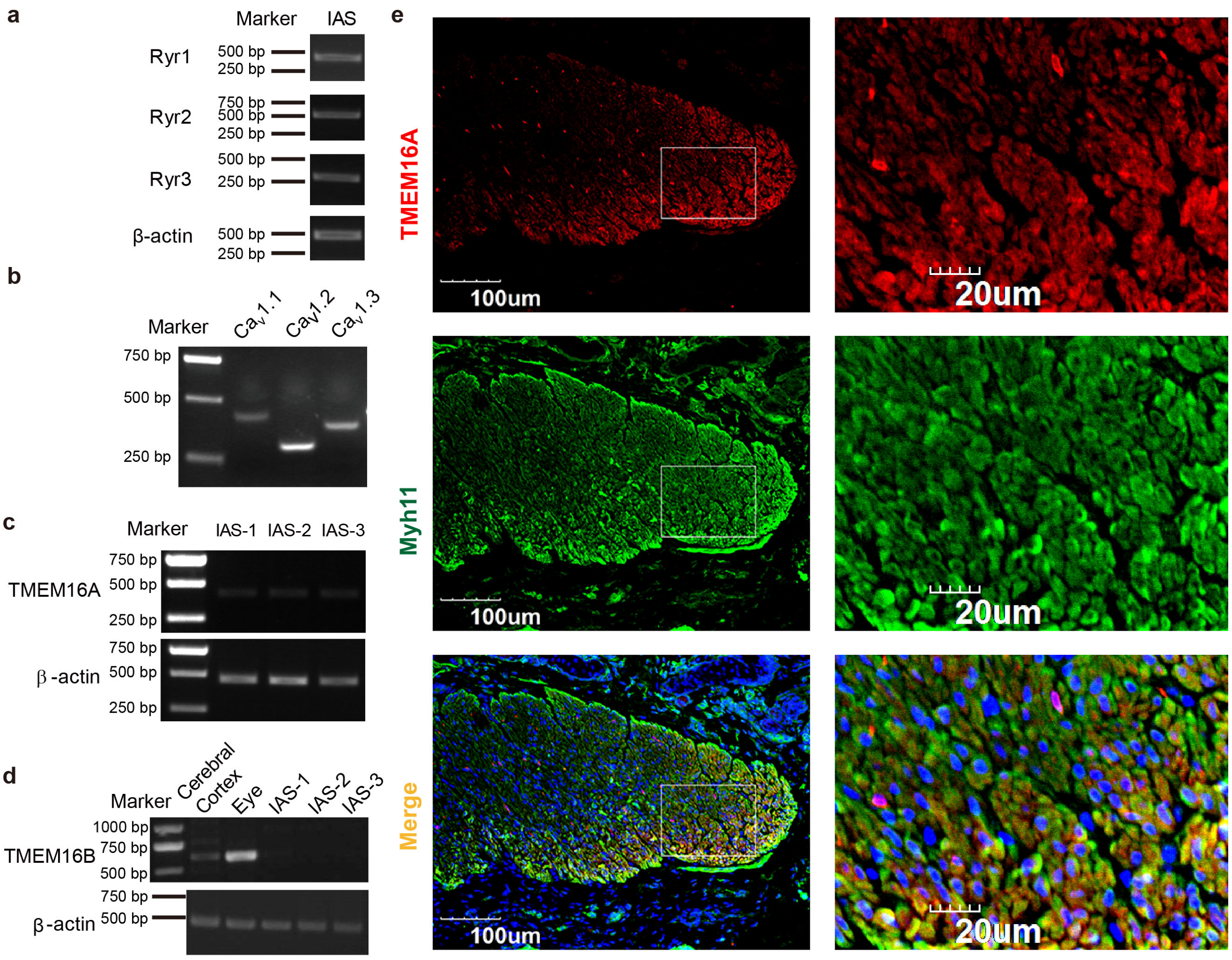

Supplementary Figure 10. Molecular characterization of the Ca^{2+} signaling module in IAS. (a) RT-PCR detected the transcripts of Ryr1, Ryr2 and Ryr3 in IAS. 3 repeats gave a similar result. (b) RT-PCR revealed the expression of L-type calcium channels Cav1.1, Cav1.2 and Cav1.3 in IAS. 3 repeats showed a similar result. (c) & (d) RT-PCR results indicate that TMEM16A (c) but not TMEM16B (d) is expressed in IAS. n=3. (e) Immunohistochemistry of TMEM16A in transverse sections of IAS smooth muscle tissue. Red represents TMEM16A and green represents Myh11, a specific marker of smooth muscle cells. DAPI was used for nuclear staining (blue). Right panels are enlarged views of the boxed region on the left images to show that TMEM16A and Myh11 are highly colocalized (yellow). Scale bars in left panels: 100 μm, bars in right panels: 20μm.

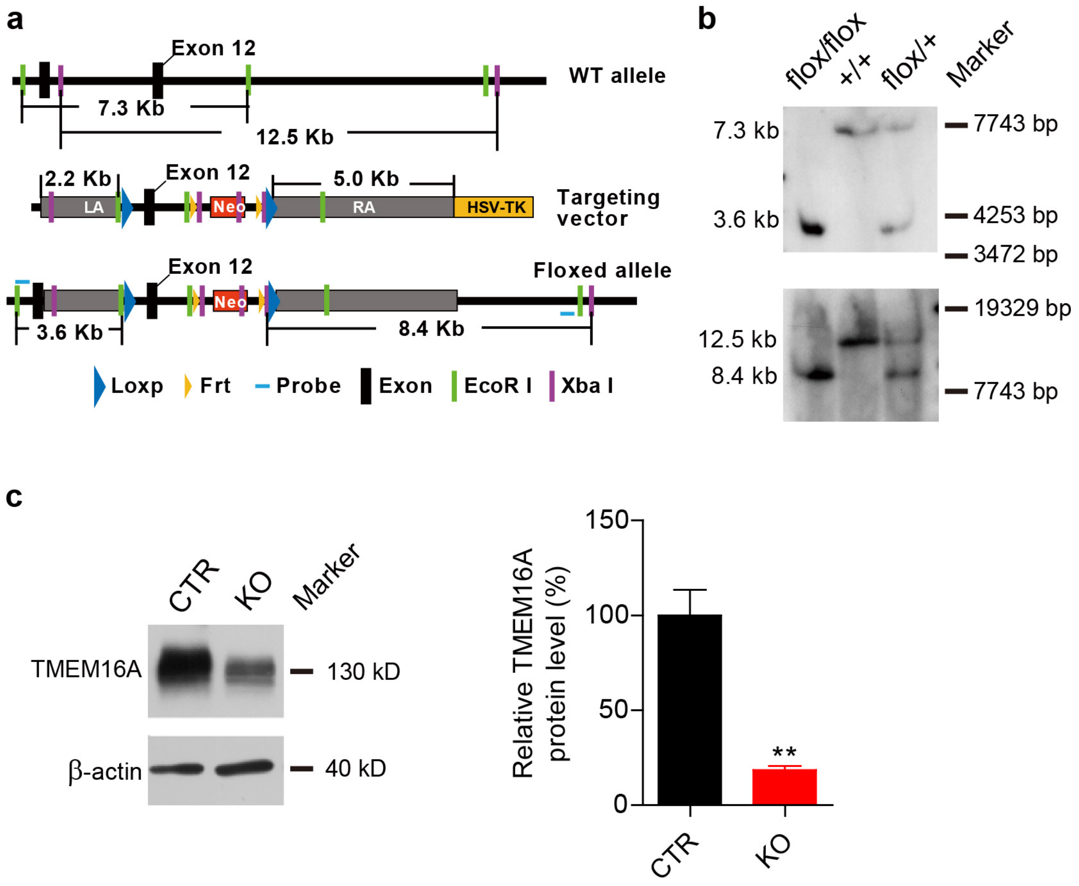

Supplementary Figure 11. Deletion of TMEM16A in IAS smooth muscle. (a) Schematic representation of the *Tmem16a* floxed allele strategy. A floxed *Neo* cassette was targeted upstream of exon 12, and excision of the floxed *Neo* cassette left behind a single loxP site at the targeted locus. A single PGK-*Neo* cassette, flanked by FRT sites and a downstream loxP site, was then introduced downstream of exon 12. The EcoR I site ahead the first loxP site and the Xba I site in the Neo cassette were used for Southern blot analysis. The floxed allele (*Tmem16a*^{flox}) was formed after homologous recombination in ES cells. (b) Southern blot analysis of liver DNA isolated from homozygous (flox/flox) floxed, wild-type (+/+), and heterozygous (flox/+) floxed mice after digestion with EcoR I or Xba I. The wild-type and floxed allele yield, respectively, 7.3-kb and 3.6-kb fragments by EcoR I digesting, and 12.5-kb and 8.3-kb fragments by Xba I digesting. (c) Western blot analysis of TMEM16A protein in IAS from *Tmem16a*$^{flox/+}$; *SMA-Cre*$^{+}$ (CTR) and *Tmem16a*$^{flox/flox}$; *SMA-Cre*$^{+}$ (KO) mice. The amount of TMEM16A was normalized by β-actin. n=6. Bars represent mean ± s.e.m., ***P*< 0.01 by two-tailed student's *t*-test.

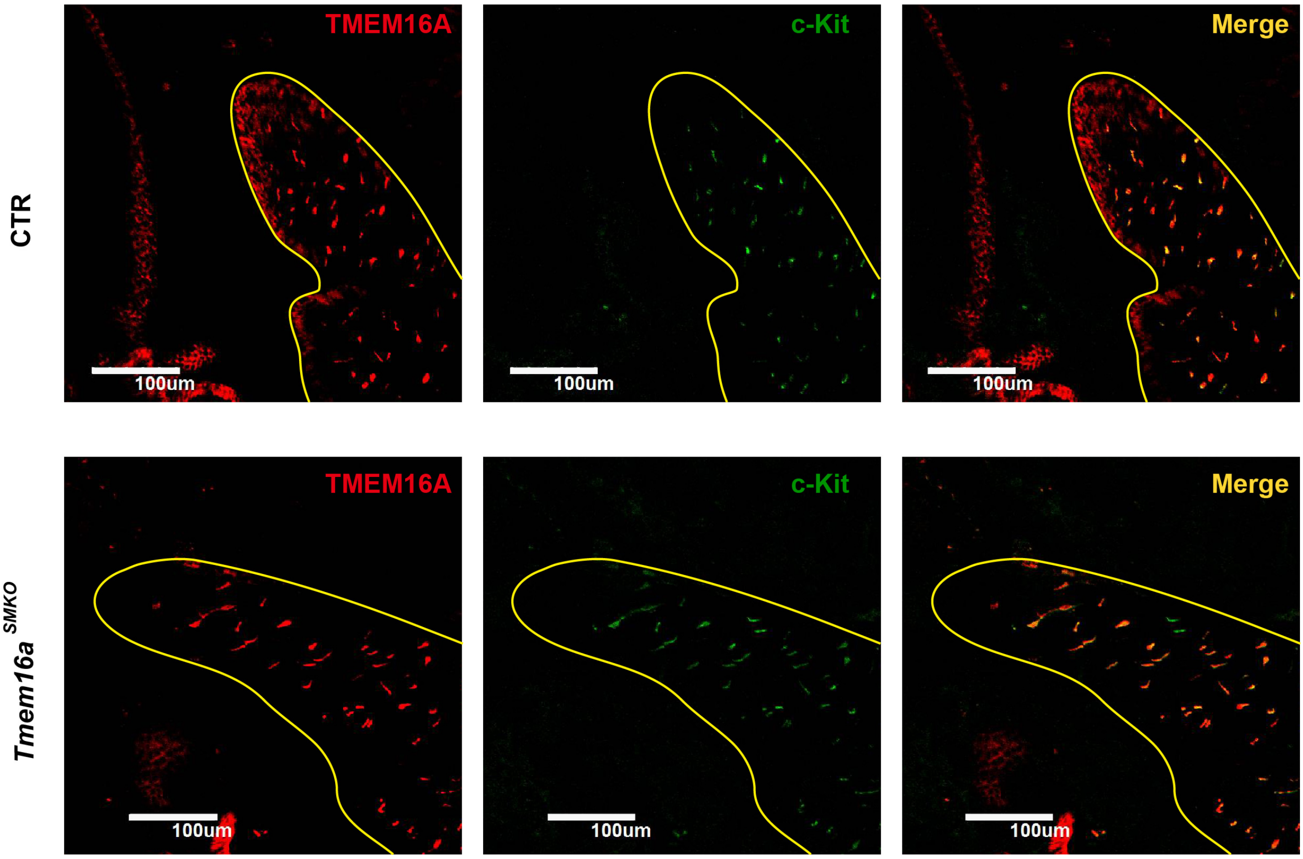

Supplementary Figure 12. TMEM16A expression in the interstitial cells of Cajal (ICCs) of mouse. Red represents TMEM16A and green c-Kit, a specific marker of ICCs. Note that in the IAS from *Tmem16a*^{SMKO}, TMEM16A staining in ICCs are preserved, indicating the specificity of TMEM16A deletion in IAS smooth muscle cells.

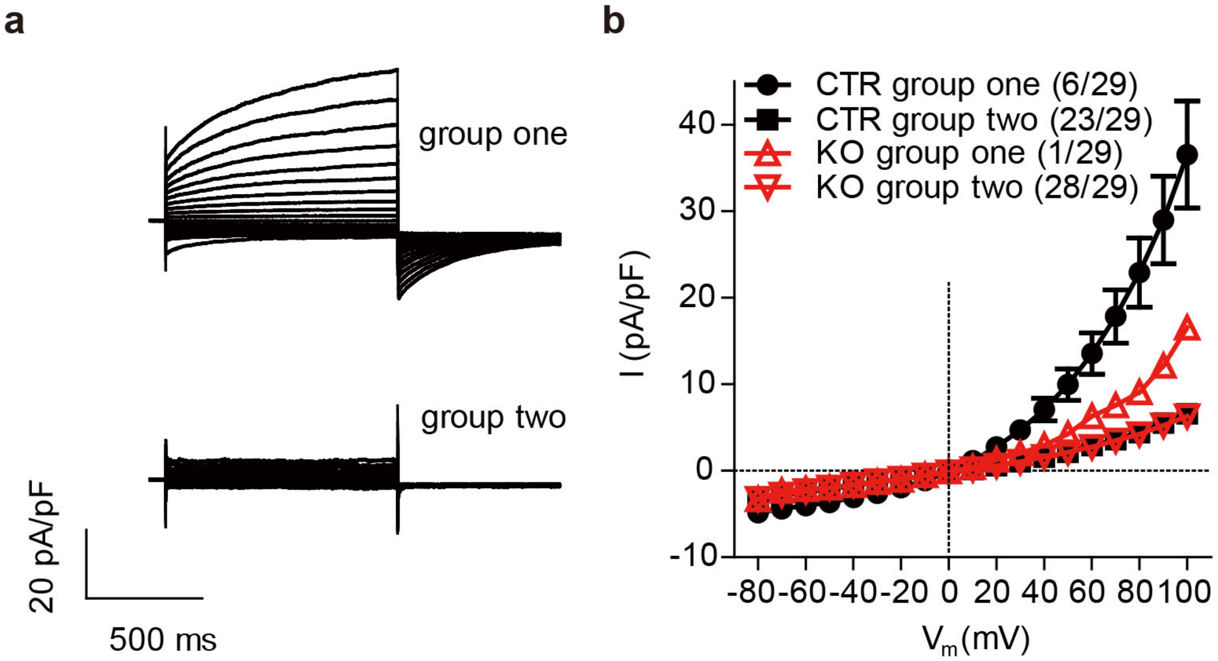

Supplementary Figure 13. Comparison of Cl_{Ca} currents (original traces on the left and mean values on the right) in IAS-SMCs from CTR and *Tmem16a* KO mice. (a). Whole cell currents recorded under conventional whole cell configuration in response to 1s voltage pulses from -80 to +100 mV in 10mV increments followed by 700ms pulses to -60mV, in freshly isolated IAS-SMCs. The cytosolic $[Ca^{2+}]_i$ was set at 600 nM dialyzed via patch pipette, and holding potential was 0 mV. (b) Current-voltage relationship as estimated by the currents at the end of test pulses. Note that essentially none of the IAS-SMCs from *Tmem16a* KO mice produced Cl_{Ca} currents under this recording condition, while 6 out of 29 IAS-SMCs from CTR mice generated robust Cl_{Ca} currents, while the remaining 23 did not give rise of detectable Cl_{Ca} currents. The lack of Cl_{Ca} currents in some cells could be due to the rundown of Cl_{Ca} channels under conventional whole cell recording mode, as often occurs in native smooth muscle cells[3]. The mechanism of the rundown of Cl_{Ca} channel remains elusive. Given that TMEM16A interact/bind with a large number of proteins in the cytosol[4], it is likely that loss of one or multiple binding partners under conventional whole cell configuration contributes to the rundown.

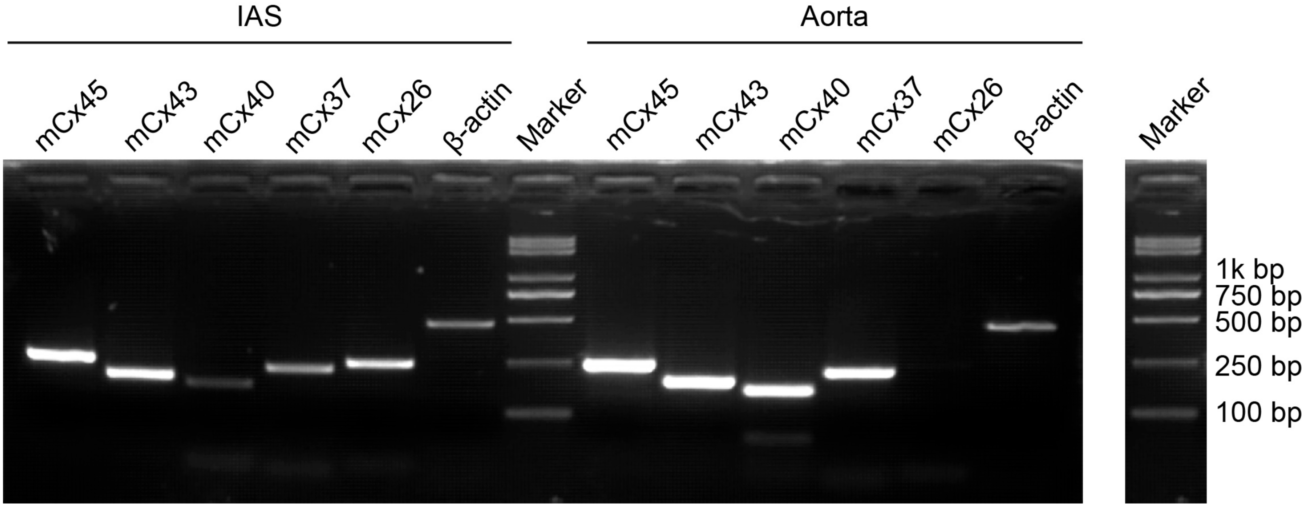

Supplementary Figure 14. Multiple gap junction protein transcripts are present in mouse IAS tissue. Aorta was used as a positive control[5, 6]. The RT-PCR primers for mouse Cx45, Cx43, Cx40, Cx37 and Cx26 are listed in Supplementary Table 2.

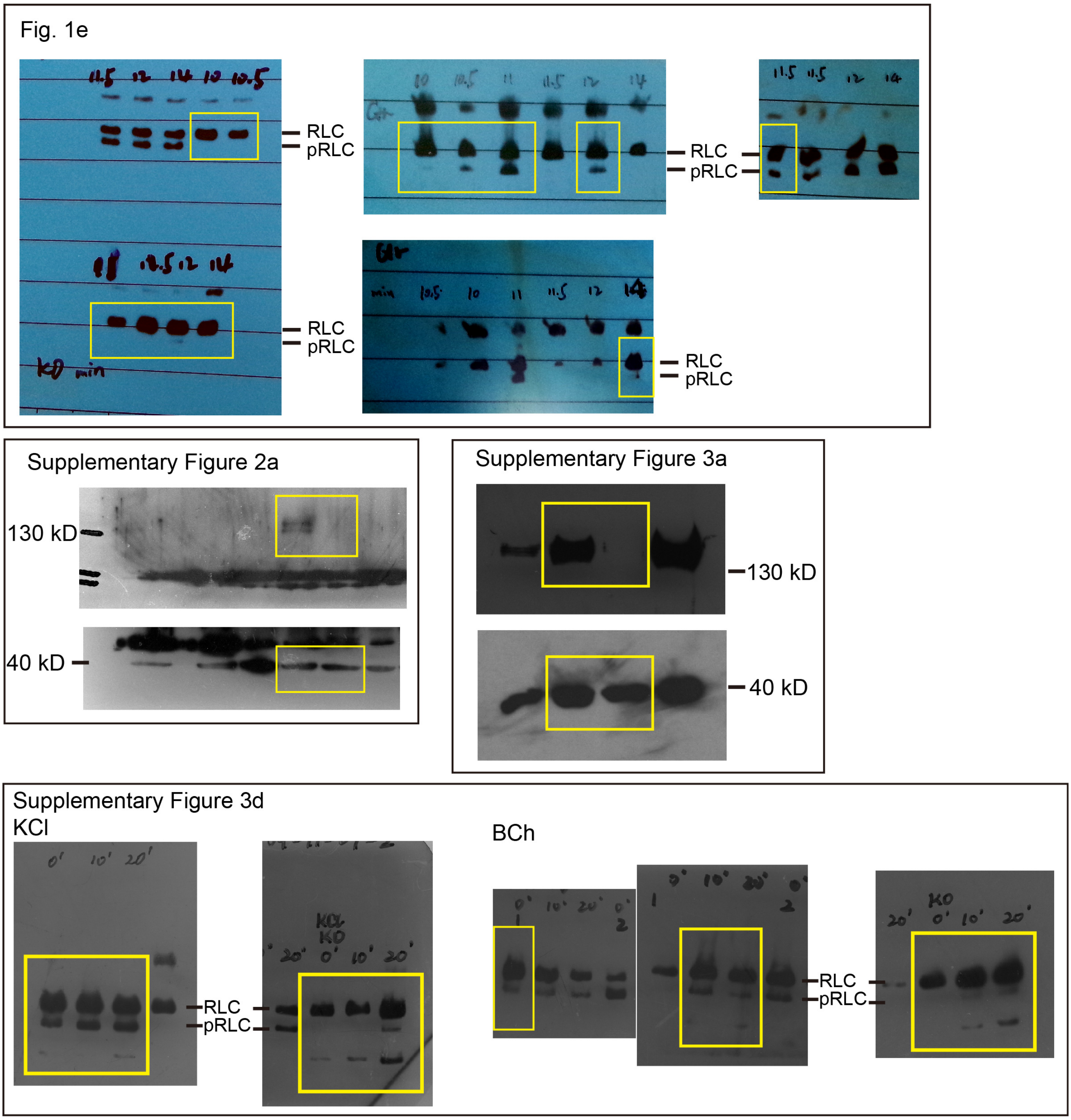

Supplementary Figure 15. Source data, western blots 1

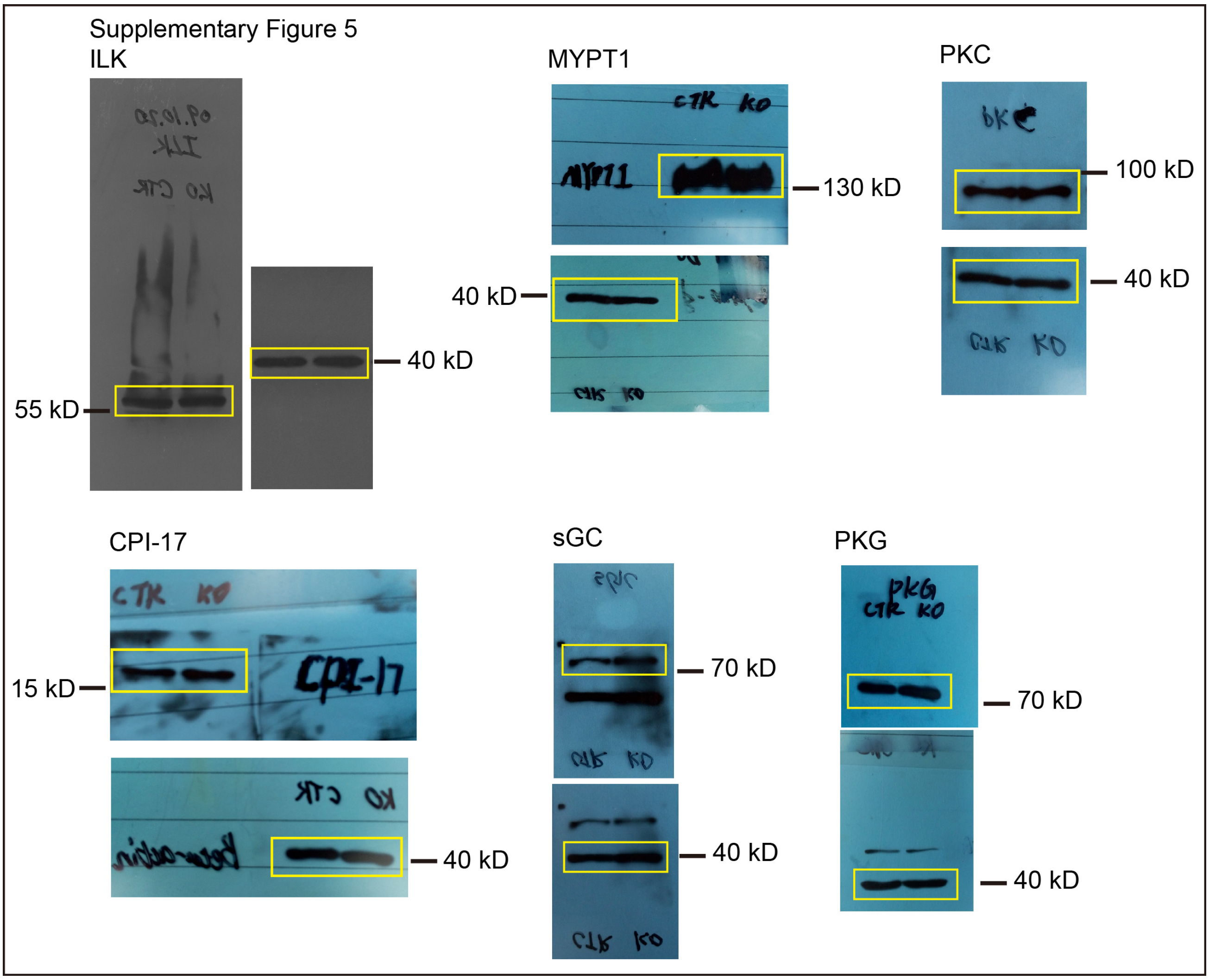

Supplementary Figure 16 Source data, western blots 2

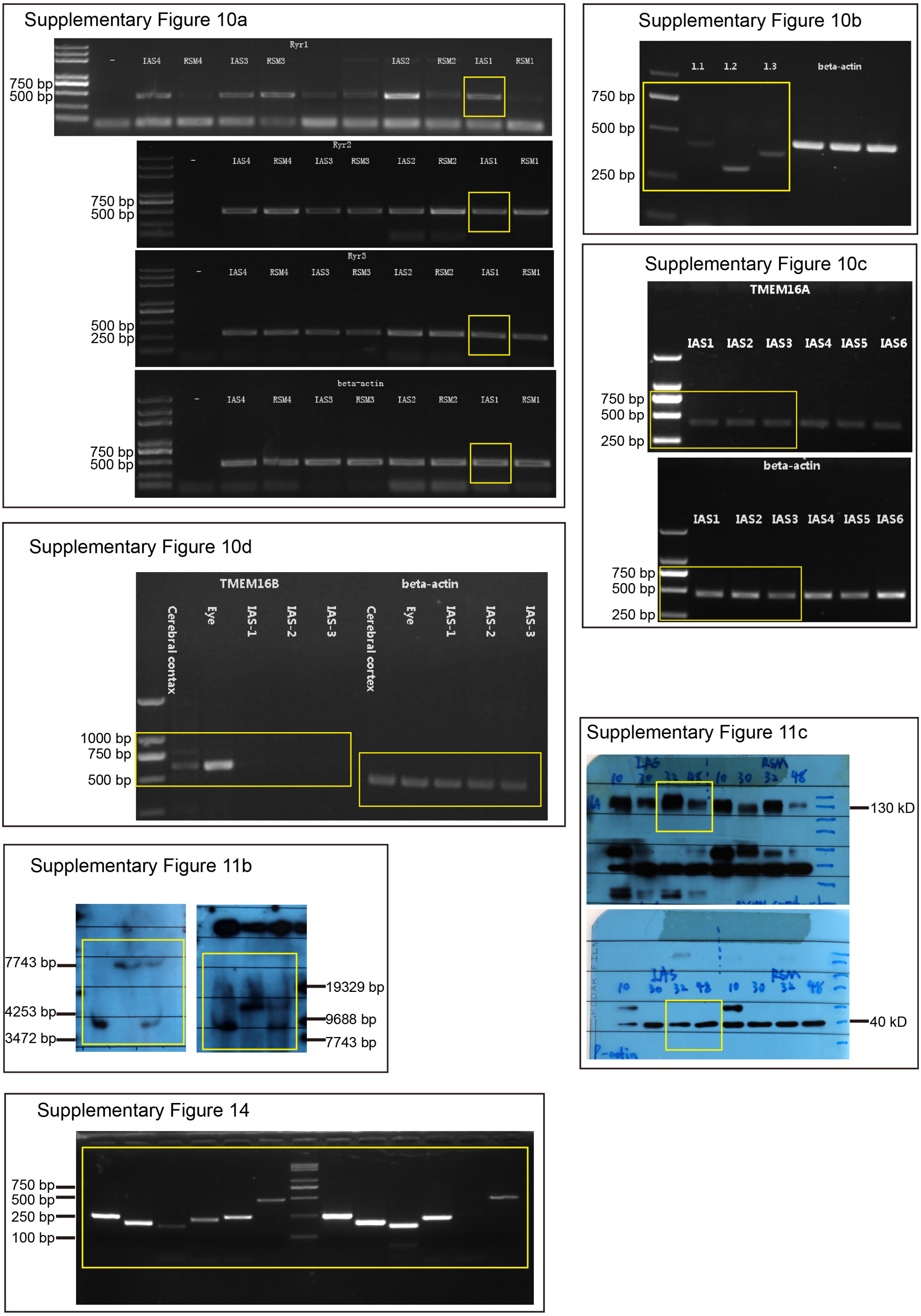

Supplementary Figure 17 Source data, RT-PCR, southern blot and western blot

Supplementary Table 1: Western blot primary antibodies

Catalog No.	Concentration	Target	Host Species	Vendor
ab64161	1:5000	RLC	Rabbit	abcam
ab53212	1:500	TMEM16A	Rabbit	abcam
07-672	1:2500	MYPT1	Rabbit	upstate
M7905 (clone K36)	1:10000	MLCK	Mouse	Sigma
I0783 (clone 65.1)	1:2000	ILK	Mouse	Sigma
05-983 (clone M110)	1:1000	PKC	Mouse	upstate
ab32213	1:2500	CPI-17	Rabbit	abcam
160897	1:1000	sGC	Rabbit	Cayman
KAP-PK005	1:1000	PKG	Rabbit	Stressgen
A5441 (clone AC-15)	1:10000	β-actin	Mouse	Sigma

Supplementary Table 2: RT-PCR Primers

Gene	Forward	Reverse
Ryr1	CATCACCTTTTTCTTCTT CGTTATC	CAGTGGAGAAGGCAC TTGAGG
Ryr2	CGATACTGCTAAAGTG ACCAACA	ATTCAATACAGCAGAC AAGGAGC
Ryr3	TTGGCAATGACTACTT TGACACG	AAGTGTAAAAGACGG ACTCTGTTG
Tmem16a	CAATCACACGCTCTCTT CCTTCAAT	GGTTGTGGGGCTGTG GTTGTTA
Tmem16b	CACAACGGGACACTACA TGG	CTTAAGCCAGTTCCCA GCAG
Actb (β-actin)	ACTGCCGCATCCTCTTC CTC	AAGCACTTGCGGTGC ACGA
Cav1.1	CAGCAGAGGAGGAAC TGGAGAG	ATCGGTCTTTTCAGTTT GTCCAC
Cav1.2	GAGACGACAGCCTGTA GTGGGAG	ACAAAAGGTAAGAGGG TGCCGT

Cav1.3	TCCTACCCCACCATCC CATC	AGTCAGACTAGCTGGC GTGAAA
Connexin26 (mCx26)	CAAGCCGATTTTGTCT GCAAC	CGATACGGACCTTCTGG GTTT
Connexin37 (mCx37)	CCCACATCCGATACTG GGTG	CGAAGACGACCGTCCTC TG
Connexin40 (mCx40)	CCACATTCGTTATTGG GTACTGC	TACTGGGTACTCATAGG CACC
Connexin43 (mCx43)	GGATCGCGTGAAGGG AAGAAG	TTGCGGCAGGAGGAATT GTTT
Connexin45 (mCx45)	AGATCCACAACCATTC GACATTT	TCCCAGGTACATCACAG AGGG

Supplementary References:

1 Zhuge R, et al. Ca^{2+} spark sites in smooth muscle cells are numerous and differ in number of ryanodine receptors, large-conductance K^+ channels, and coupling ratio between them. *Am. J. Physiol. Cell Physiol.* **287**, C1577-1588 (2004).

2 Paez-Cortez, J. et al. A new approach for the study of lung smooth muscle phenotypes and its application in a murine model of allergic airway inflammation. PLoS One **8**, e74469 (2013).

3 Angermann, J. E. *et al.* Mechanism of the inhibition of Ca^{2+}-activated Cl^- currents by phosphorylation in pulmonary arterial smooth muscle cells. *J. Gen. Physiol.* **128**, 73-87, (2006).

4 Perez-Cornejo, P. *et al.* Anoctamin 1 (Tmem16A) Ca^{2+}-activated chloride channel stoichiometrically interacts with an ezrin-radixin-moesin network. *Proc. Natl Acad. Sci. U. S. A.* **109**, 10376-10381, (2012).

5 van Kempen, M. J. & Jongsma, H. J. Distribution of connexin37, connexin40 and connexin43 in the aorta and coronary artery of several mammals. *Histochem. cell Biol.* **112**, 479-486 (1999).

6 Ko, Y. S., Coppen, S. R., Dupont, E., Rothery, S. & Severs, N. J. Regional differentiation of desmin, connexin43, and connexin45 expression patterns in rat aortic smooth muscle. *Arterioscl. Throm. Vas.* **21**, 355-364 (2001).
